# Noise Modelling and Comparison of P(VDF-TrFE) and PZT-5A Piezoelectric Hydrophones for Thermoacoustic Sensing

**DOI:** 10.3390/s26216679

**Published:** 2026-10-22

**Authors:** Elia Arturo Vallicelli, Mattia Tambaro, Marcello De Matteis

**Affiliations:** 1 Department of Physics, University of Milano-Bicocca, 20126 Milano, Italy; mattia.tambaro@unimib.it (M.T.); marcello.dematteis@unimib.it (M.D.M.); 2 Section of Milano-Bicocca, National Institute for Nuclear Physics (INFN), 20126 Milano, Italy

**Keywords:** piezoelectric hydrophone, noise-equivalent pressure, PVDF, PZT, Mason model, KLM model, charge amplifier, photoacoustic, ionoacoustic, thermoacoustic

## Abstract

Thermoacoustic signals, as in photoacoustic imaging or ionoacoustic range verification, are of a few millipascals, and the noise-equivalent pressure (NEP) of receiver and readout decides whether they are detectable. This work presents a frequency-domain noise model of immersed piezoelectric hydrophones read by a charge amplifier, applied to a P(VDF-TrFE) copolymer and a PZT-5A ceramic of identical geometry. A distributed Mason/KLM model gives the charge sensitivity, the matching layer and, via the Nyquist theorem at its electrical port, the thermal noise of the thickness mode while a multi-branch network adds the in-plane modes. Over 1 kHz–1 MHz ionoacoustic bandwidth the NEP is 47.6 mPa rms for the copolymer and 123.7 for PZT-5A. As sensors they are within 0.3 dB; the 8.3 dB difference comes from the ceramic capacitance, which multiplies the amplifier voltage noise. A k-Wave study of a 200 MeV proton beam at 54 mGy per pulse gives signal-to-noise ratios of 30.5 and 22.1 dB and range errors of 0.06 and 0.33 mm.

## 1. Introduction

The noise characteristics of piezoelectric acoustic transducers and of their readout electronics set the sensitivity floor of a wide class of signal-limited experiments. In fluid-coupled geometries the noise-equivalent pressure (NEP) of the detection chain is determined by the interplay of four principal mechanisms: the acoustic thermal noise of the surrounding fluid, Johnson–Nyquist noise arising from dielectric losses in the piezoelectric material, flicker noise of the readout amplifier at low frequencies, and the broadband electronic noise of the analogue front end [1,2,3,4]. The element is coupled to the water through its radiation resistance, and by the fluctuation–dissipation theorem that coupling is necessarily accompanied by a stochastic pressure on the element face. The same mechanism that allows the element to receive the acoustic signal obliges it to receive the thermal fluctuations of the medium. This contribution cannot be reduced by any choice of readout electronics [2,3,4].

Several application domains are limited directly by transducer NEP. In photoacoustic imaging the maximum optical fluence is constrained by tissue safety standards, so the detectable signal amplitude is bounded and the minimum detectable pressure is formally the NEP of the detection chain [5,6,7,8,9]. Piezoelectric polymers have become materials of choice for wideband photoacoustic receivers owing to their low acoustic impedance, mechanical flexibility and intrinsically broad bandwidth [10,11]. Focused P(VDF-TrFE) elements are now also made optically transparent, so that the excitation light reaches the tissue through the receiver itself [12]. In bubble-chamber dark matter detectors, piezoelectric sensors record the acoustic emission of nuclear-recoil-induced bubbles, and the frequency-resolved sensitivity of the sensor governs the discrimination between WIMP-like recoils and alpha-particle backgrounds [13,14]. In underwater metrology and cavitation monitoring, polymer hydrophones calibrated to IEC 62127-1 [15] serve as broadband reference receivers, and their NEP determines the minimum detectable pressure in passive cavitation detection.

**Figure 1:**
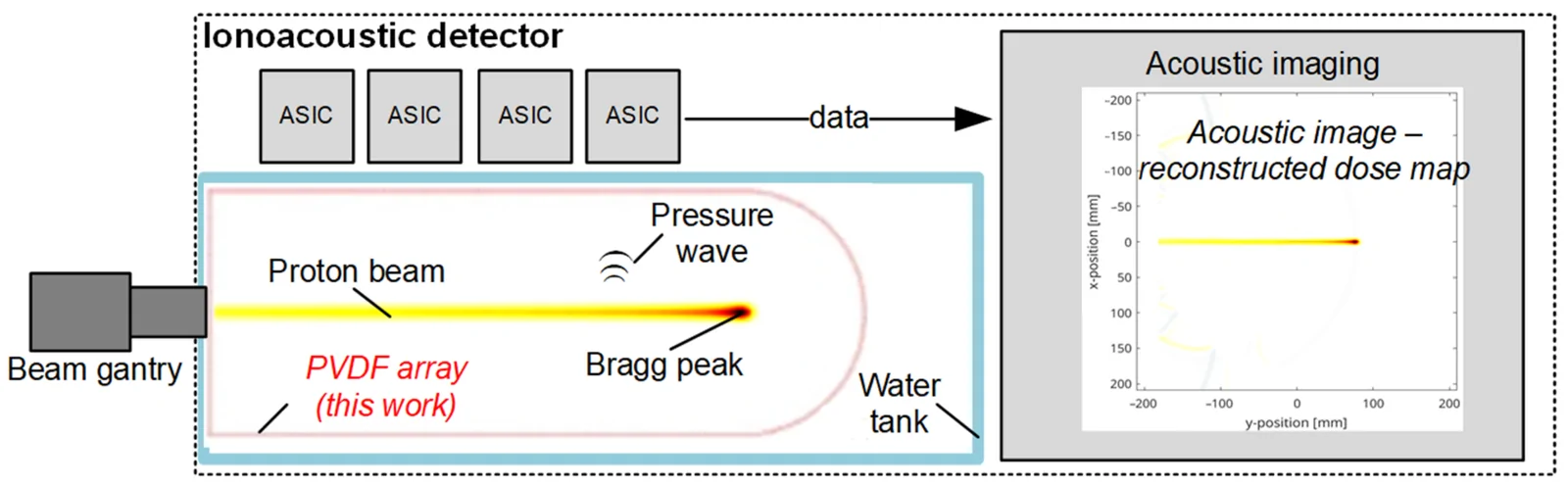
Generic block scheme of an ionoacoustic detector, showing the proton beam energy deposition in water, the acoustic sensor array (red), the analogue front end (ASIC) and the acoustic imaging stages.

A particularly stringent application is ionoacoustics, in which the thermomechanical stress induced by a pulsed ion beam generates an acoustic wave whose time of flight encodes the Bragg-peak position, with direct relevance to real-time range verification in hadron therapy [16,17,18,19,20,21,22,23,24,25,26]. The mechanism is the same with pulsed X-rays. X-ray-induced acoustic computed tomography exploits it for imaging [27], down to three-dimensional absorption images from a single projection [28], and ionising radiation acoustic imaging has visualised in real time the dose delivered to a patient with liver metastases during radiotherapy [29]. At clinically relevant conditions, that is a 200 MeV proton beam delivering from a few to a few tens of mGy per pulse, and the acoustic amplitude at a surface detector is of the order of tens of millipascals. This places the measurement at the sensor noise floor and makes the transducer NEP the primary determinant of whether Bragg-peak localisation is feasible at therapeutic dose levels [30,31]. The same constraint drives the search for other receivers. A millimetre-sized optical hydrophone has detected the Bragg-peak signal in water with a higher signal-to-noise ratio than a piezoelectric hydrophone of 29 mm diameter [32], and bulk piezoelectric and opto-mechanical micromachined detectors have been compared directly for optoacoustic sensing [33]. Such comparisons mean little unless they are made on the noise-equivalent pressure, readout included. Figure 1 shows the detection chain considered in this work, from the energy deposition to the reconstructed image. We have previously shown that careful noise engineering of a polymer-based analogue front-end ASIC yields a marked improvement of the signal-to-noise ratio at ionoacoustic frequencies, that is 30–500 kHz for 60–200 MeV beams [31,34,35,36]. This motivates a physically grounded, frequency-dependent noise model for immersed piezoelectric elements.

The present work has two objectives. The first is methodological. The charge sensitivity of an immersed piezoelectric plate is very often written as the product of the piezoelectric charge constant, the active area and a transmission coefficient *α* = 2*Z_w_*/(*Z_w_* + *Z_c_*). Section 2.3 examines the conditions under which that expression holds. It is exact for an element free to strain laterally and facing a medium that behaves as a half-space. Neither condition is met by the receiver considered here: within a one-dimensional thickness-mode description the lateral strains are suppressed, so that the appropriate material constant is the clamped combination *e*_33_/*c*_33_*^E^*, not the free *d*_33_. In addition, *α* as written is the transmission coefficient of particle velocity, while the stress in an acoustically thin plate is imposed by its terminations and not by the material impedance. The two departures act in opposite directions. The clamped constant is smaller than *d*_33_, and the transmitted stress is larger than *α* implies. For a polymer whose impedance is close to that of water both effects are moderate and the net difference is about 5 dB. For a stiff ceramic the second prevails, and the sensitivity is underestimated by 14 to 17 dB across the thermoacoustic band. Since the expression is often used to compare materials, the discrepancy falls where it matters most.

The second objective is the comparison itself, performed with the distributed formulation for two elements of identical geometry, a 3 × 25 mm active area immersed in water: a P(VDF-TrFE) 75/25 copolymer, which is the material of the receivers we employ experimentally [34], and a PZT-5A ceramic with a quarter-wave matching layer and a damped backing. The transducer is described at two complementary levels, set out in Section 2.4. A distributed-parameter Mason/KLM model provides the electromechanical response and represents the matching layer as a transmission-line section. The same model gives the thermal noise of the thickness mode, through the Nyquist theorem applied at its electrical port, so that signal and noise are referred to the same incident pressure. A multi-branch Butterworth–Van Dyke network adds the in-plane extensional modes and keeps track of every dissipation mechanism, so that each contributes its thermal noise once and only once. The model accounts for seven noise sources and separates sensor-intrinsic from electronics-limited NEP through a noise-figure formulation. It is then applied to the ionoacoustic detection of a clinical 200 MeV proton beam, quantifying both the signal-to-noise ratio available at therapeutic pulse doses and the range error contributed by the transducer response itself.

This is a modelling study, and no new measurements are reported. The model is verified instead against analytical limits, across its two levels of description and for its sensitivity to the least well-constrained parameters, as described in Section 3.2. The limitations that follow from this are stated in Section 4.4.

## 2. Materials and Methods

### 2.1. Transducer Geometry and Material Properties

Two bulk piezoelectric elements were modelled and compared: a poled P(VDF-TrFE) 75/25 copolymer film and a PZT-5A ceramic. Both were assumed to have a rectangular active area of *W* = 3 mm × *H* = 25 mm (*A* = 75 mm^2^), immersed in water (*ρ_w_* = 1000 kg m^−3^, *c_w_* = 1500 m s^−1^, *Z_w_* = 1.5 MRayl). The target thickness-mode resonance was *f*_0_ = 1 MHz, so that for each material the thickness follows from the half-wavelength condition *t* = *c_D_*/(2*f*_0_) and is not a free parameter. The lateral dimensions correspond to the element pitch and aperture of the ionoacoustic arrays that motivate this study [34,36], in which the long dimension provides aperture along the beam axis while the narrow dimension sets the transverse sampling. Material constants are summarised in Table 1 and are taken from manufacturer data and from the IEEE Standard on Piezoelectricity [37,38,39,40].

The resulting lateral-to-thickness ratios, W/t = 1.38 for PZT-5A and 2.61 for the copolymer, are well below the value of about ten conventionally required for a clean, decoupled thickness mode. The in-plane extensional modes thus fall inside the frequency range of interest, and they are treated explicitly in Section 2.5, where they also carry the lateral compliance that a purely one-dimensional description leaves out (Section 2.3).

The acoustic boundary condition on the rear face differs between the two materials, each being modelled in the configuration in which it is used in practice. The copolymer element is water-backed, as in the immersed receivers previously reported by the authors [34]. The PZT-5A element is backed by a tungsten-loaded epoxy of acoustic impedance 6 MRayl, which is standard practice for damped ceramic receivers [41], and carries the quarter-wave matching layer of Section 2.4 on its front face. The backing is an acoustic termination and therefore a dissipative element: its thermal noise is included in the radiation term of Section 2.6, and it also raises the transmitted stress and hence the charge sensitivity, as shown in Section 2.3.

### 2.2. Static Capacitance with Fringe-Field Correction

The clamped capacitance *C*_0_ of the rectangular element was computed with a first-order fringe-field correction following the Kirchhoff edge-field approximation [42]:

(1) $$C_{0}=\frac{\varepsilon_{33}^{S}A}{t}\left[1+\frac{2t}{\pi W}\ln\left(\frac{\pi W}{2t}\right)+\frac{2t}{\pi H}\ln\left(\frac{\pi H}{2t}\right)\right]$$

where the two additional terms approximate the fringe contribution along each pair of edges. The correction is accurate to within a few per cent for *t*/min(*W*,*H*) ≤ 0.5; for the PZT element (*t*/*W* ≈ 0.73) it slightly overestimates *C*_0_, and a finite-element evaluation would be required for a more accurate result. The correction increases the parallel-plate estimate by 52% for PZT-5A and 45% for the copolymer, giving *C*_0_ = 384.5 pF and 6.90 pF respectively. This ratio of 56 is the single most consequential difference between the two devices, and it propagates into the readout design of Section 2.6 and into the noise budget of Section 3.1.

### 2.3. Charge Sensitivity of an Immersed Plate

The quantity that converts an incident pressure into the input signal of the charge amplifier is the short-circuit charge sensitivity *S_q_*(*f*) = |Q/P|, since the amplifier holds the element at virtual ground. A quasi-static expression of the form

(2) $$S_{q}=d_{33}A\;\alpha ,\;\alpha =\frac{2Z_{w}}{Z_{w}+Z_{c}}$$

is in widespread use as a first estimate of hydrophone sensitivity. Equation (2) is an exact result within its own hypotheses: an element free to strain laterally, so that the stress state within it is uniaxial, and an interface problem in which the receiving medium may be treated as a half-space and the transmitted quantity of interest is the particle velocity [37,43]. The receiving configuration modelled here meets neither hypothesis, for two independent reasons, and a different expression is required.

First is the mechanical boundary condition, which selects the material constant. Short-circuiting the electrodes makes the field in the plate vanish, *E*_3_ = 0. This by itself does not fix the constant. In the strain-charge form the constitutive relations then give *D*_3_ = *d*_33_ *T*_3_ + *d*_31_ (*T*_1_ + *T*_2_), while in the stress-charge form they give *D*_3_ = *e*_33_ *S*_3_ + *e*_31_ (*S*_1_ + *S*_2_). These are the same physics written in different variables, and the choice between them is made by the lateral boundary condition, not by the electrical one [37]. Under uniaxial stress, *T*_1_ = *T*_2_ = 0, the free constant *d*_33_ applies. Under uniaxial strain, *S*_1_ = *S*_2_ = 0, one has *T*_3_ = *c*_33_*^E^ S*_3_, so that *D*_3_ = (*e*_33_/*c*_33_*^E^*) *T*_3_. The one-dimensional transmission-line description used in Section 2.4 is, by construction, a uniaxial-strain model: the lateral degrees of freedom do not exist within it, so the constant consistent with it is the clamped combination *e*_33_/*c*_33_*^E^* [44,45,46]. This is not a claim that the plate is rigidly clamped by its mounting. The lateral compliance that *d*_33_ embodies is retained in the model. It is carried by the in-plane branches of Section 2.5, where it appears as resonances at their own frequencies instead of a static enhancement of the thickness-mode constant; inserting *d*_33_ into the one-dimensional branch would count that compliance a second time. For PZT-5A, *e*_33_/*c*_33_*^E^* = 144 pC/N against *d*_33_ = 374 pC/N, and for the copolymer, 29 pC/N against 30 pC/N. The separation is large for the ceramic, whose electromechanical coupling is strong, and negligible for the polymer, for which the two descriptions almost coincide.

Second is the transmission factor, which is set by the acoustic thickness of the element. The ratio 2*Z_w_*/(*Z_w_* + *Z_c_*) is the transmission coefficient of particle velocity at a water–material interface, and it is the appropriate factor when the receiving medium behaves as a half-space; the corresponding coefficient for stress is 2*Z_c_*/(*Z_w_* + *Z_c_*), which for PZT-5A is 1.91, not 0.085 [43,46]. Neither is the relevant limit in the present case, because in the thermoacoustic band the element is acoustically thin: at 100 kHz the wavelength is 15 mm in water and 43 mm in the ceramic, against a plate thickness of 2.17 mm, about one twentieth of the wavelength in the ceramic. A thin plate does not present a half-space to the incident wave. The stress within it is imposed by its two terminations, and the quasi-static limit becomes

(3) $$S_{q}\to \left(\frac{e_{33}}{c_{33}^{E}}\right)A\frac{2Z_{b}}{Z_{w}+Z_{b}}$$

where *Z_b_* is the rear termination. Equation (3) is the low-frequency limit of the distributed model of Section 2.4 and agrees with it at 1 kHz to within 0.2% in all the configurations examined: for PZT-5A with the 6 MRayl backing it gives 17.3 fC/Pa against 17.3 fC/Pa computed, for the same element water-backed 10.8 against 10.8, and for the water-backed copolymer 2.17 against 2.17. At higher frequency the mass reactance of the plate is no longer negligible against its terminations. For the ceramic at 100 kHz it amounts to *ωρ*t = 10.6 MRayl, against *Z_w_* + *Z_b_* = 7.5 MRayl, and the sensitivity falls to 13.5 fC/Pa without the matching layer and to 12.8 fC/Pa with it; for the copolymer *ωρ*t = 1.3 MRayl and the sensitivity stays at 2.18 fC/Pa.

The effect on a material comparison is not uniform, which is why the distinction has practical consequences. The two departures act in opposite directions for both materials. The clamped constant lowers the sensitivity, by 8.3 dB for PZT-5A and by 0.3 dB for the copolymer, while the stress set by the terminations raises it with respect to *α*, by 25.5 dB and 5.5 dB respectively. For the copolymer the net effect is moderate: 1.20 fC/Pa from Equation (2) against 2.18 fC/Pa from the distributed model, a difference of 5.2 dB. For PZT-5A the transmission term prevails: 2.39 fC/Pa against 12.8 fC/Pa at 100 kHz, an underestimate of 14.6 dB, and of 14–17 dB across 30–200 kHz. Since the input-referred noise scales as 1/*S_q_*, applying the quasi-static expression to both materials and comparing the results returns an apparent polymer advantage of 18.2 dB over the processing band, against 8.6 dB from the distributed model. About 10 dB of that figure reflects the unequal validity of the expression for the two materials, not a property of the materials themselves. All results reported in Section 3 use the distributed model.

The output voltage of the charge amplifier follows as

(4) $$S_{v}\left(f\right)=\frac{S_{q}\left(f\right)}{C_{f}}H_{HP}\left(f\right),\;H_{HP}\left(f\right)=\frac{j\omega R_{f}C_{f}}{1+j\omega R_{f}C_{f}}$$

with a −3 dB low-frequency corner at *f_low_* = 1/(2π*R_f_C_f_*). Three sensitivities must be distinguished. The intrinsic charge sensitivity *S_q_* is a property of the element and of its acoustic terminations. The open-circuit voltage sensitivity *S_q_*/*C*_0_ additionally involves the element capacitance and, because of the two-orders-of-magnitude difference in permittivity, reverses the ordering: at 100 kHz it is 33.4 µV/Pa for PZT-5A against 316 µV/Pa for the copolymer. The charge-amplifier output sensitivity *S_q_*/*C_f_* is a property of the readout design, since *C_f_* is chosen by the designer, and must not be read as a material figure of merit. The comparison in this work is made at the level of the input-referred noise. There *C_f_* cancels between signal and noise in every term except two, the amplifier voltage noise, which is referred to the input through *C*_0_ + *C_in_* + *C_f_* (Section 2.6.4), and the feedback resistor, whose value follows from *C_f_*. Since *C_f_* is set to *C*_0_/10 (20 dB gain) for both elements, this residual dependence is nearly the same for the two and does not bias the comparison.

### 2.4. Distributed Model, Matching Layer and the Two Levels of Description

Two representations of the same transducer are used. They are frequently presented as alternatives, most recently in a review of the Mason, KLM and Butterworth–Van Dyke circuits for medical ultrasound transducers [47], so this section sets out why both are required.

The distributed-parameter model, in the Mason formulation and equivalently in that of Krimholtz, Leedom and Matthaei [44,48,49], describes the element as an acoustic transmission line of thickness t and characteristic impedance *ρc_D_*·A, terminated by the front and rear acoustic loads and coupled to an electrical port through the force factor Φ = *e*_33_A/t. It is exact for the thickness mode at every frequency, it resolves the odd harmonic resonances at 3*f*_0_ and 5*f*_0_ that fall within the 15 MHz range considered here, it places no restriction on *k_t_*, and, decisively for the present work, it admits additional transmission-line sections so that the water–matching layer–ceramic–backing stack is represented with its interface reflections and phase shifts intact. What it cannot represent is motion that is not one-dimensional, in particular the in-plane extensional modes that the aspect ratios of Section 2.1 place inside the band of interest. In the receiving configuration used here the incident field is represented by its Thévenin equivalent, a blocked force 2PA in series with the source impedance *Z_w_*A, and the electrical port is short-circuited, so that the computed quantity is the short-circuit charge sensitivity of Section 2.3 [43,45,46,50]. The same port also delivers the thermal noise of every dissipation that loads the thickness mode, and the electrical impedance *Z_e_* seen from it is what sets that noise (Section 2.6.1).

The lumped Butterworth–Van Dyke network describes the same element as a static capacitance in parallel with one motional branch per vibrational mode. It has two roles here. The first is to carry the in-plane extensional modes, which the one-dimensional model cannot represent, each with its own motional resistance and therefore its own thermal noise. The second is bookkeeping: each dissipation mechanism appears in the network as one identified resistance, so that it can be checked that each contributes to the noise budget once and only once. This is what makes the loss accounting of Section 2.7 verifiable.

**Figure 2:**
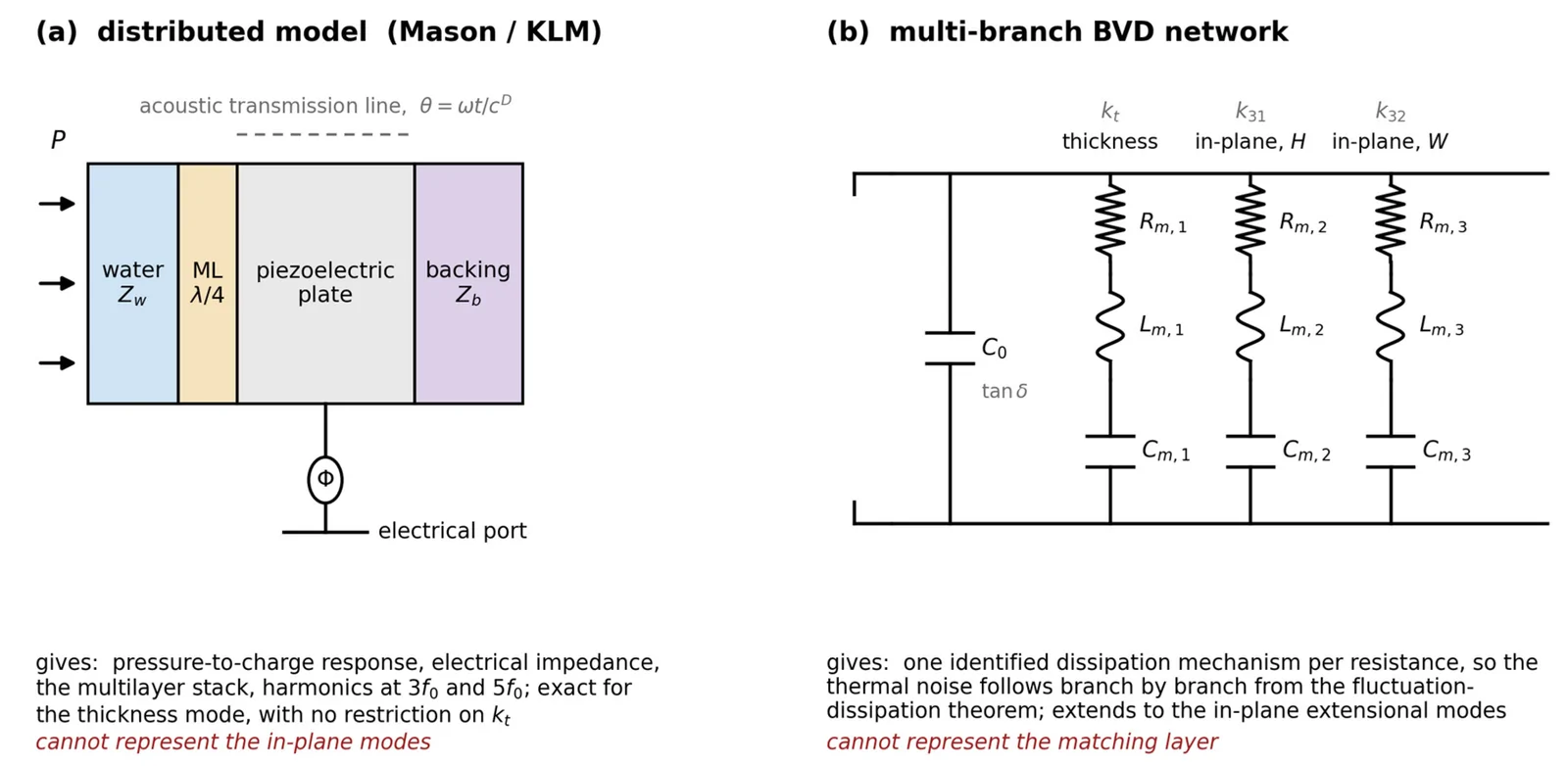
The two levels at which the transducer is described. (**a**) Distributed-parameter Mason/KLM model: the piezoelectric plate is an acoustic transmission line terminated by the front and rear loads, with the quarter-wave matching layer represented as a further transmission-line section, and coupled to the electrical port through the force factor Φ. The arrows on the left indicate the incident acoustic pressure *P*. (**b**) Multi-branch Butterworth–Van Dyke network: the static capacitance *C*_0_ with its dielectric loss, in parallel with one motional branch per mode, the thickness mode and the two in-plane extensional modes. Each resistance corresponds to one identified dissipation mechanism and therefore to one thermal noise term.

The two levels of description are illustrated in Figure 2. The distributed model provides the charge sensitivity, the electrical impedance, the matching-layer stack and the thermal noise of the thickness mode, while the multi-branch network provides the in-plane modes and the bookkeeping of the losses. The thickness branch of the network is not used to compute the noise. It serves as a cross-check of the impedance, and Section 3.2 shows where the lumped description stops being adequate.

For PZT-5A a quarter-wave matching layer is bonded to the front face [51,52]. The impedance giving maximum power transfer at *f*_0_ is *Z_ML_* = √(*Z_c_Z_w_*) = 7.11 MRayl, and is the value adopted. The broadband criterion of Desilets et al. [51] would give (*Z_c_Z_w_*^2^)^1/3^ = 4.23 MRayl, which trades peak transfer for bandwidth, and since the ionoacoustic band lies well below resonance the distinction has little effect on the results. The layer is modelled as an alumina-loaded epoxy with *c_ML_* = 2700 m/s, implied density *ρ_ML_* = *Z_ML_*/*c_ML_* = 2633 kg/m^3^, quarter-wave thickness *t_ML_* = *c_ML_*/(4*f*_0_) = 0.675 mm and mechanical quality factor *Q_m,ML_* = 20 [53]. The effective acoustic load presented to the front face follows from the lossy transmission-line transformation

(5) $$Z_{eff}\left(f\right)=Z_{ML}\frac{Z_{w}+jZ_{ML}\tan\left(k^{\prime }t_{ML}\right)}{Z_{ML}+jZ_{w}\tan\left(k^{\prime }t_{ML}\right)}$$

with k′ = (*ω*/*c_ML_*)(1 − j/2*Q_m,ML_*). In contrast to a lumped treatment, in which the influence of the layer is absorbed into an effective quality factor evaluated at *f*_0_, *Z_eff_*(*f*) is applied here as the frequency-dependent front termination of the distributed model. The reflections and phase shifts at the water–ML and ML–ceramic interfaces are represented across the whole band. The same layer also transforms the blocked force on the front face, which becomes *F_oc_* = 2PA/[cos(k′*t_ML_*) + j(*Z_w_*/*Z_ML_*) sin(k′*t_ML_*)], so that termination and source are transformed together. This is not a refinement of detail. A lumped network has no node at which a layer can be inserted, so within it the matching layer can only appear as additional damping, which adds radiation noise without the corresponding improvement in acoustic coupling, so that the layer appears to degrade the noise-equivalent pressure where the distributed treatment shows it to improve it.

The loaded mechanical quality factor of the thickness mode, evaluated in the mechanical domain as *Q_mech_* = *ω*_0_*m_eff_*/(*R_rad_* + *R_int_*) with *m_eff_* = *ρ*At/2, *R_rad_* = (Re *Z_eff_* + *Z_b_*)A for the matched ceramic and (*Z_w_* + *Z_b_*)A for the water-loaded polymer, and *R_int_* = *ω*_0_*m_eff_*/*Q_m_*, is 1.5 for the backed ceramic and 1.8 for the water-backed copolymer. The acoustic terminations are plane-wave impedances, which holds when the wavelength is small compared with the element dimensions. For the present geometry the equivalent radius is a = √(A/π) = 4.89 mm, so that ka = 1 at 48.9 kHz in water. For a baffled circular piston of the same area the radiation resistance normalised to *ρ*cA is 1 − *J*_1_(2ka)/ka [43]. It equals 0.42 at ka = 1 and stays below unity up to ka = 1.92, that is 94 kHz. Below that frequency the plane-wave value overestimates both the radiation damping and the associated thermal noise, while the blocked force of the incident wave is unchanged, so the treatment is conservative there. Above it the piston value exceeds the plane-wave one by at most 13%, near 125 kHz, which amounts to 0.5 dB on the radiation term. The quality factors quoted above, evaluated at *f*_0_ where ka ≈ 20, are unaffected.

### 2.5. Multi-Branch Network and In-Plane Modes

The electrical impedance of each element was modelled with a multi-branch Butterworth–Van Dyke network, that is the static capacitance *C*_0_ with its parallel dielectric loss shunted by one series motional branch (*R_m,i_*, *L_m,i_*, *C_m,i_*) per mode:

(6) $$Z_{e}\left(f\right)={\left[j\omega C_{0}\left(1-j\tan\delta \right)+\sum_{i}\frac{1}{R_{m,i}+j\omega L_{m,i}+\frac{1}{j\omega C_{m,i}}}\right]}^{-1}$$

The motional capacitance of each branch was approximated as *C_m,i_* = 8ki^2^*C*_0_/[π^2^(1 − *k_i_*^2^)]. For the thickness branch the motional resistance *R_m_* = *R_mi_* + *R_mw_* combines an internal term *R_mi_* = √(*L_m_*/*C_m_*)/*Q_m_* and a radiation term *R_mw_* = (*Z_w_* + *Z_b_*)A/Φ^2^, with Φ = *e*_33_A/t and *e*_33_ = *k_t_*√(*ρc_D_*^2^*ε^S^*). This branch serves only the comparison of impedances in Section 3.2, and the thermal noise of the thickness mode is taken from the distributed model (Section 2.6.1).

A single motional branch represents the thickness mode alone, which for the aspect ratios of Section 2.1 is insufficient. The in-plane extensional resonances are set by the in-plane dimensions and by the bar velocity *c_bar_* = (*ρs*_11_*^E^*)^−1/2^, equal to 2805 m/s for PZT-5A and 1179 m/s for the copolymer, giving f = *c_bar_*/2L. The corresponding frequencies are 56.1 kHz and 467.5 kHz for PZT-5A along the 25 mm and 3 mm dimensions, and 23.6 kHz and 196.4 kHz for the copolymer. They depend on the in-plane dimensions alone, cannot be displaced by changing the thickness, and all lie within or close to the ionoacoustic band.

The quality factor of the lateral branches is taken to be that of the internal damping alone, and radiation loading from the element edges is neglected. This choice has less influence than it appears. The mean-square current of a series branch is *k_B_*T/*L_m_* by equipartition, independently of its resistance, so that the quality factor determines the shape of each resonance but not the contribution of the branch to the integrated budget, which is set by *L_m_* and hence by the transverse coupling coefficient and the static capacitance.

The distinction between the two mechanical loss mechanisms does matter for the noise. Radiation damping is reciprocal with the acoustic field, so a mode that radiates weakly also receives weakly and the two effects largely cancel in the input-referred budget. Internal damping is not reciprocal: it dissipates without coupling to the acoustic field and contributes noise without a corresponding signal path. Because the lateral branches are dominated by internal damping they are a noise term and not a sensitivity term. Their coupling to a normally incident plane wave is not determined by a one-dimensional model, and the results reported below adopt the conservative assumption that they contribute dissipation, and hence noise, but no signal, which bounds their effect from above.

### 2.6. Charge Amplifier, Feedback Network and Noise Sources

A transimpedance charge amplifier was assumed as the readout. The feedback capacitance was set to *C_f_* = *C*_0_/10, giving a noise gain *G_n_* = 1 + (*C*_0_ + *C_in_*)/*C_f_* of 11.0 for PZT-5A and 11.7 for the copolymer, where *C_in_* = 0.5 pF is the input capacitance of an integrated front end. The bias resistor follows from the required low-frequency cut-off *f_low_* = 100 Hz as *R_f_* = 1/(2π*f_low_C_f_*), that is 41.4 MΩ for PZT-5A and 2.31 GΩ for the copolymer. The amplifier noise parameters, *e_n_* = 5 nV/√Hz and *i_n_* = 1 fA/√Hz with flicker corners of 200 Hz and 50 Hz respectively, are design values. They are representative of an integrated charge amplifier of the class developed by the authors for ionoacoustic readout [31,34,35,36], not the measured figures of a specific device, and Section 3.2 examines how the results depend on them.

Realising the copolymer feedback network is the principal practical difficulty of the polymer solution. On standard FR4, surface leakage and humidity limit the achievable resistance to roughly 10–100 MΩ, which would raise the low-frequency corner by more than an order of magnitude. Substrates of low surface conductivity such as PTFE or Rogers, together with a guard ring driven at the inverting-input potential, are required to sustain gigaohm values. Alternatively a CMOS switch in the feedback path realises an equivalent resistance of several gigaohms at the cost of a periodic reset, which injects charge through the gate–drain overlap capacitance and samples the switch thermal noise onto the feedback capacitor. This gives √(*k_B_*T/*C_f_*) = 77.5 µV rms at *C_f_* = 0.69 pF against 10.4 µV for PZT-5A. Both contributions are constant during the acquisition that follows a given reset and are removed by correlated double sampling. In ionoacoustic operation this requires no additional hardware, because the acquisition is gated by the accelerator trigger and the acoustic signal arrives after a time of flight of tens of microseconds. The subtraction of two samples taken Δt apart acts as a high-pass response 2|sin(πfΔt)|, which suppresses the reset pedestal and the flicker component while leaving the 30–500 kHz signal band unaffected, at the cost of a factor √2 on the white component above 1/Δt.

The third route is an integrated front end. With *C_f_* = 0.69 pF the feedback capacitance is comparable to the parasitic capacitance of a printed-circuit board. A parasitic across the feedback path adds directly to *C_f_* and reduces the charge gain, while one at the summing node adds to *C*_0_ and raises the noise gain. The input capacitance of the amplifier itself acts in the same way, and is not negligible for the polymer. A discrete JFET-input operational amplifier with *C_in_* ≈ 10 pF would raise the noise gain from 11.7 to 25.5 and the integrated NEP from 47.6 to 56.7 mPa rms, that is by 1.5 dB. For PZT-5A the same capacitance changes the noise gain from 11.0 to 11.3 and the NEP by 0.2 dB, so that the advantage of the copolymer falls to 6.9 dB. In this configuration an application-specific integrated front end is a requirement and not an optimisation, and this motivates the ASIC implementations previously reported by the authors [31,34,36]. The same argument applies to the rest of the acquisition chain. The front end operates next to a clinical accelerator, so its biasing and reference blocks must tolerate the radiation environment [54], and the digitisation of its output at tens of megasamples per second is met by converters developed for particle-detector readout [55], while the subsequent decimation and filtering are performed in the digital domain [56].

Environmental stability deserves a final remark. The low-frequency corner depends on the product *R_f_C_f_*, and *R_f_* realised either as a discrete high-value resistor or as a leakage-limited CMOS switch is strongly temperature dependent. Leakage typically doubles every 8 to 10 K, which halves *R_f_* and doubles *f_low_*, and surface humidity acts in the same direction. In a deployed hydrophone the design value of *f_low_* is thus a lower bound on the actual corner, unless the front end is conformally coated and temperature stabilised, which again favours an integrated implementation.

Seven independent noise sources were included. Four are intrinsic to the element, namely acoustic radiation into the water and the backing, internal mechanical damping, the in-plane extensional modes and dielectric loss; three belong to the readout, namely the amplifier voltage and current noise and the Johnson noise of the feedback resistor. All are expressed as power spectral densities at the charge-amplifier output and are subsequently divided by *S_v_*(*f*)^2^ to obtain the input-referred equivalent pressure noise.

#### 2.6.1. Acoustic Radiation Noise

The thermal noise of the thickness mode is obtained from the distributed model itself, by applying the Nyquist theorem at its electrical port [2,3,4]. With the electrodes held at virtual ground, the port delivers a short-circuit noise current of power spectral density 4*k_B_*T·Re(1/*Z_e_*), where *Z_e_* is the electrical impedance of Section 2.4 with all its terminations in place. *Z_e_* is computed with a lossless capacitance, so it contains the mechanical dissipation only and the dielectric loss of Section 2.6.3 is not counted twice. The amplifier integrates this current onto *C_f_* exactly as it integrates the signal charge, which gives an output contribution 4*k_B_*T·Re(1/*Z_e_*)·|*H_HP_*|^2^/(*ωC_f_*)^2^. Every dissipation of the thickness mode, whether radiation into the water through the matching layer, radiation into the backing or internal loss, thus reaches the output through its own transfer. Since *Z_e_* and *S_q_* come from the same network, noise and signal are referred to the same incident pressure by construction. The radiation part is the conductance of the same plate computed with a lossless material, *Q_m_* → ∞. For a face radiating into water the floor is *k_B_*T*Z_w_*/A per unit incident plane-wave pressure, which is the thermal noise of the medium reduced by the directivity of the aperture [45]. It amounts to 9.10 µPa/√Hz for the 75 mm^2^ aperture considered here. A thin plate loaded by water on both faces receives twice that power, √(2*k_B_*T*Z_w_*/A) = 12.87 µPa/√Hz.

#### 2.6.2. Internal Mechanical Damping Noise

Structural and viscous losses within the element enter the distributed model through the complex wavenumber k = (*ω*/*c_D_*)(1 − j/2*Q_m_*). Their thermal noise is the remainder of the port conductance of Section 2.6.1, that is the difference between Re(1/*Z_e_*) for the actual material and for the lossless one, and it reaches the output through the same integration onto *C_f_*. For both materials it is a minor term, because the loaded quality factor of the thickness mode is set by the terminations and not by *Q_m_* (Section 2.4). Near the thickness resonance the lossy plate has a lower and broader peak than the lossless one, so the difference can turn negative; it is then set to zero, which overstates the thermal noise of the thickness mode by at most 0.3 mPa rms over 1 kHz–1 MHz and leaves the processing band unchanged.

#### 2.6.3. Dielectric Loss Noise

Dielectric losses are represented by a parallel conductance G = *ωC*_0_ tan *δ*, which generates a current noise 4*k_B_*T*ωC*_0_ tan *δ* at the amplifier input node; the charge amplifier integrates it onto *C_f_*, giving an output noise 4*k_B_*T·*C*_0_ tan *δ*/(*ωC_f_*^2^). When input-referred, *C_f_* cancels and the NEP depends on *C*_0_ tan *δ*/*S_q_*^2^.

The loss tangent of the polymer is the single most influential material parameter in the budget, and it is strongly frequency dependent. We adopt the measured frequency dependence reported for P(VDF-TrFE) copolymer [57], namely tan *δ* = 0.01 at 3 kHz, 0.03 at 10 kHz, 0.11 at 100 kHz and 0.22 at 1 MHz, interpolated log–log, which corresponds to a power law of exponent 0.53. An immediate consequence is that the dielectric contribution does not fall as 1/f, as would follow from a constant loss tangent, but as f^−0.47^. Because that measurement was performed on copolymer blends instead of the specific film modelled here, Section 3.2 reports the sensitivity of the comparison to a scaling of the whole curve by a factor between 0.25 and 2. For PZT-5A a constant tan *δ* = 0.020 was used [37].

#### 2.6.4. Amplifier Voltage and Current Noise

The voltage noise, including its flicker component, is *e_n_*^2^(*f*) = *e_n,w_*^2^(1 + *f_c,v_*/f) and appears at the input amplified by the noise gain, giving an output contribution *e_n_*^2^(1 + (*C*_0_ + *C_in_*)/*C_f_*)^2^. When referred to the input it becomes *e_n_*(*f*)(*C*_0_ + *C_in_* + *C_f_*)/*S_q_*(*f*), so that it grows in proportion to the element capacitance: with *C*_0_ + *C_f_* ≈ 423 pF for PZT-5A against 7.6 pF for the copolymer, and with charge sensitivities differing by a factor of six in the opposite direction, this term is the dominant asymmetry between the two devices. The current noise *i_n_*^2^(*f*) = *i_n,w_*^2^(1 + *f_c,i_*/f) is integrated by the feedback capacitance and contributes *i_n_*^2^(*f*)/(*ωC_f_*)^2^.

#### 2.6.5. Feedback Resistor Noise

The bias resistor contributes 4*k_B_*T*R_f_*/[1 + (*ωR_f_C_f_*)^2^] at the output, flat below *f_low_* and falling as 1/f^2^ above it. Referred to the input, this term and the integrated current noise of Section 2.6.4 fall as 1/f^2^ in power, while the dielectric term falls only as f^−0.47^. For the copolymer they therefore set the floor below about 2 kHz: at 1 kHz the feedback resistor contributes 197 µPa/√Hz and the current noise 76 µPa/√Hz against 120 µPa/√Hz for the dielectric loss. In the processing band both are negligible.

#### 2.6.6. In-Plane Mode Noise

Applying the fluctuation–dissipation theorem to the motional resistance of each lateral branch gives a current noise at the input node which the amplifier integrates onto *C_f_*, so that the output contribution is *Σ_i_* 4*k_B_*T·*R_m,i_*/|*Z_m,i_*(*f*)|^2^/(*ωC_f_*)^2^, with *Z_m,i_* the branch impedance.

### 2.7. Mapping of Loss Mechanisms Across the Model Domains

Because the signal path, the electrical impedance and the noise budget draw on two representations of the same device, care is required to ensure that a given dissipation mechanism is represented once and only once. Acoustic radiation into the water and into the backing is set by the terminations of the distributed model. It enters the signal response through those terminations and the noise through the port conductance of the same model (Section 2.6.1); in the network it appears as *R_mw_* = *R_rad_*/Φ^2^ and serves only the comparison of impedances. Internal mechanical damping enters the signal response through the complex wavenumber of the plate and the noise through the remainder of the same conductance (Section 2.6.2); in the network it is *R_mi_*. Dielectric loss is the parallel conductance G = *ωC*_0_ tan *δ*. It does not enter the mechanical response at all; it is excluded from the port conductance because *Z_e_* is computed with a lossless capacitance, and it contributes the noise of Section 2.6.3 alone. The in-plane branches exist only in the network and enter as described in Section 2.6.6.

Two consequences follow. First, signal and thermal noise of the thickness mode share one network, with the same terminations and the same transfer from each dissipation to the electrodes, so that they are referred to the same incident pressure by construction; the thin-plate limit of Section 3.2 verifies this. Second, each resistance contributes to the noise budget exactly once: the dielectric loss appears only as the parallel conductance, the mechanical losses do not appear in tan *δ*, and the in-plane branches, which the distributed model does not contain, are the only part of the budget taken from the network.

### 2.8. Electronics Noise Figure and Integrated NEP

The degradation of signal-to-noise ratio caused by the readout was quantified by the noise figure NF = 20·lo*g*_10_(NE*P_total_*/NE*P_sensor_*), where the two quantities are the root-mean-square equivalent pressures integrated over 1 kHz–1 MHz. A noise figure of 0 dB indicates a noiseless readout, and NF > 3 dB implies that the electronics noise equals or exceeds the intrinsic sensor noise. One caveat applies: the figure measures the electronics against the sensor and not against the signal, so that adding a sensor noise term lowers it while raising the absolute noise. Both the total NEP and the noise figure are reported throughout. The k-Wave simulation used to assess the imaging and range accuracy at system level is described in Section 3.4, together with its results.

## 3. Results

### 3.1. Noise Budget

**Figure 3:**
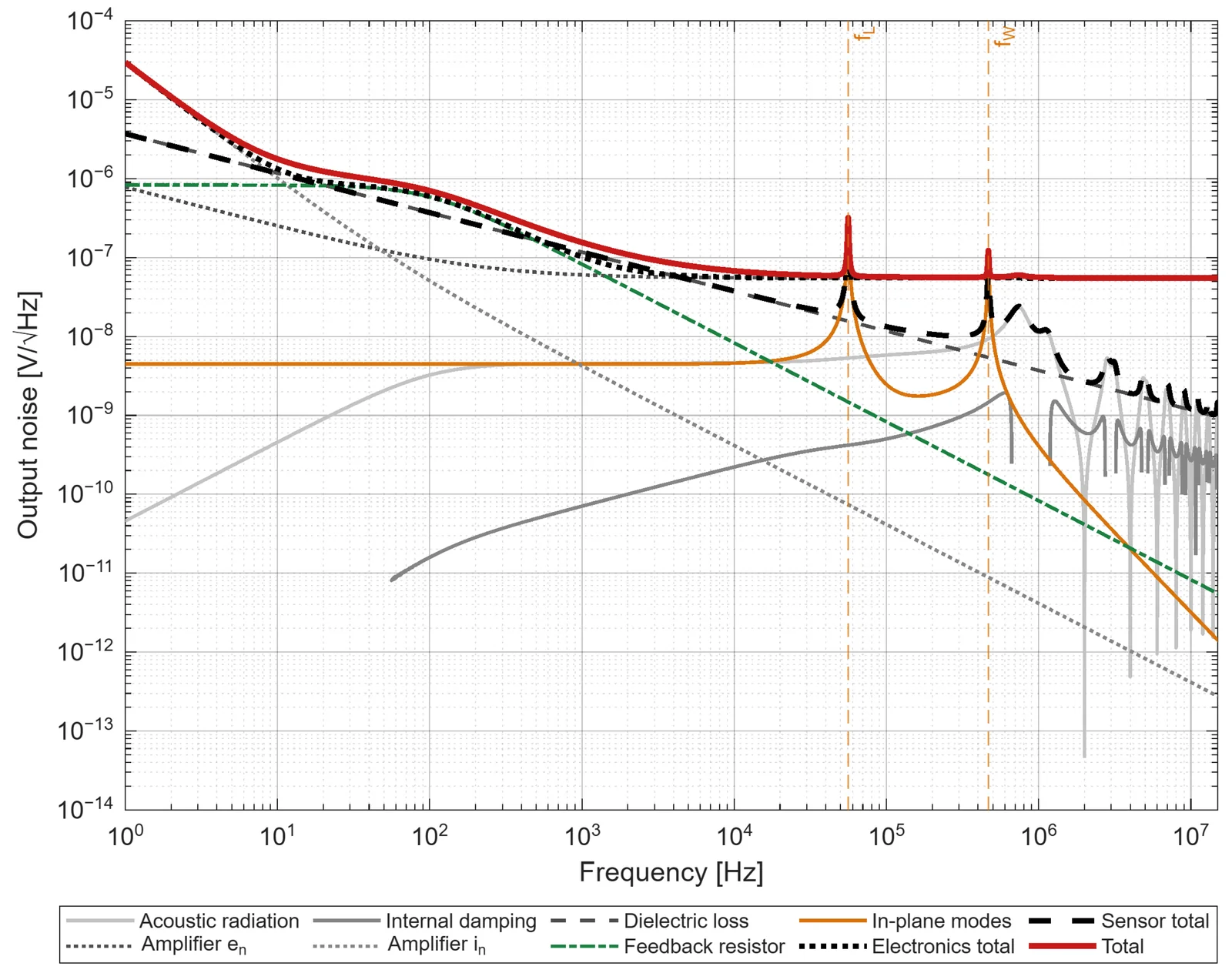
PZT-5A charge-amplifier output noise, broken down into the seven contributions of Section 2.6, with the sensor total, the electronics total and the grand total. The vertical lines mark the two in-plane extensional resonances.

**Figure 4:**
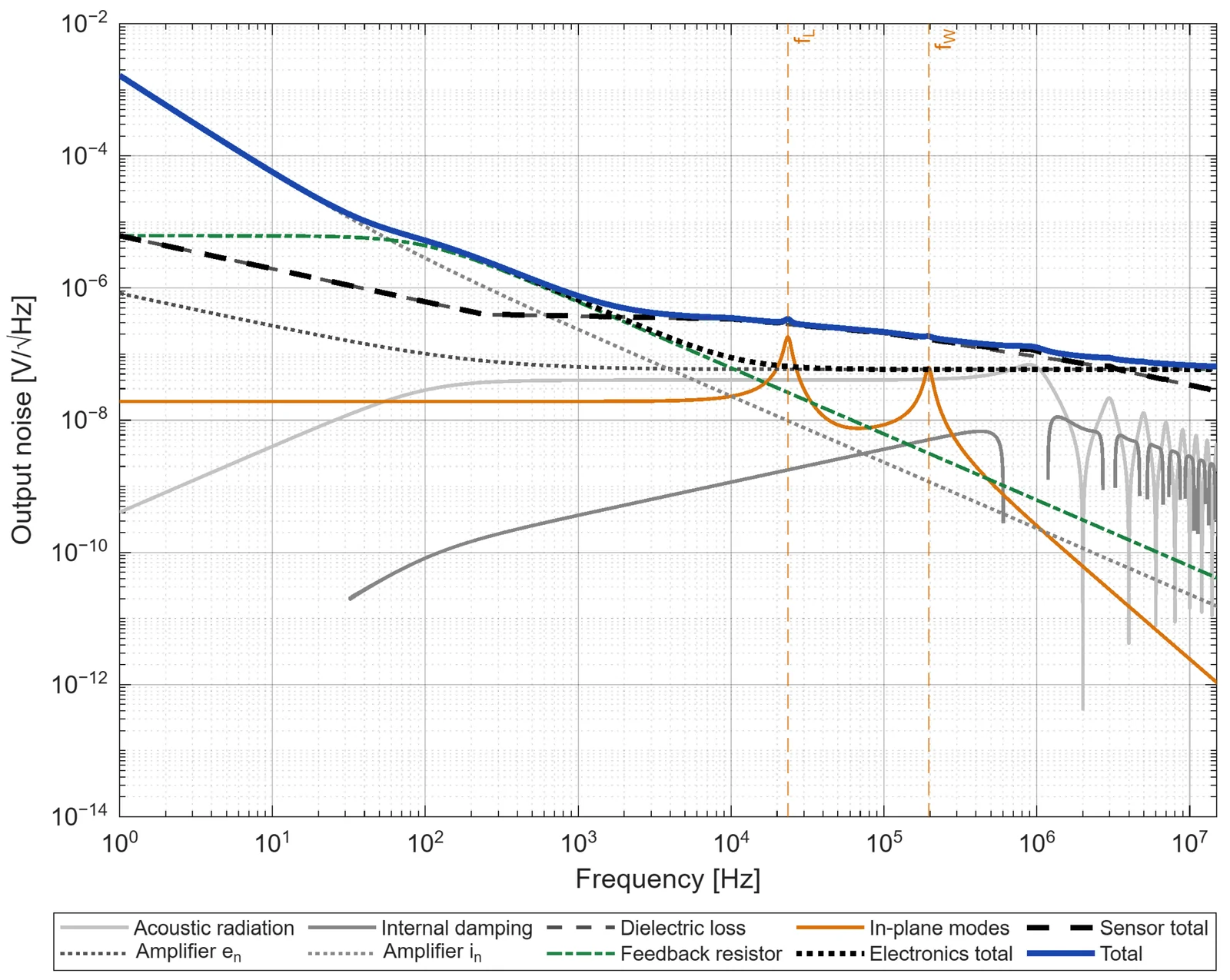
P(VDF-TrFE) charge-amplifier output noise, with the same decomposition as Figure 3.

**Figure 5:**
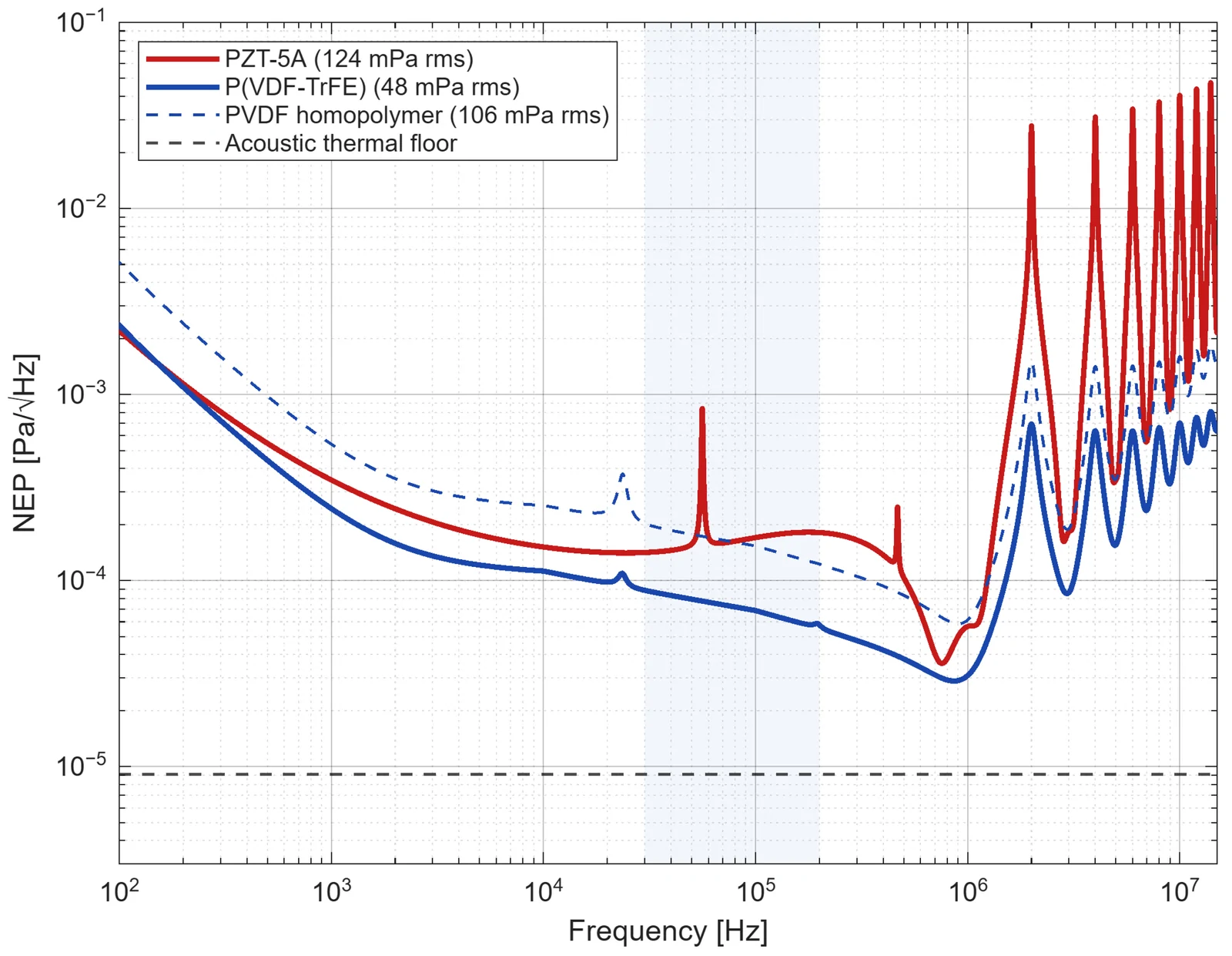
Input-referred noise-equivalent pressure of the two detectors, with the PVDF homopolymer for reference and the acoustic thermal floor of the aperture. The shaded region is the 30–200 kHz processing band of Section 3.4.

**Figure 6:**
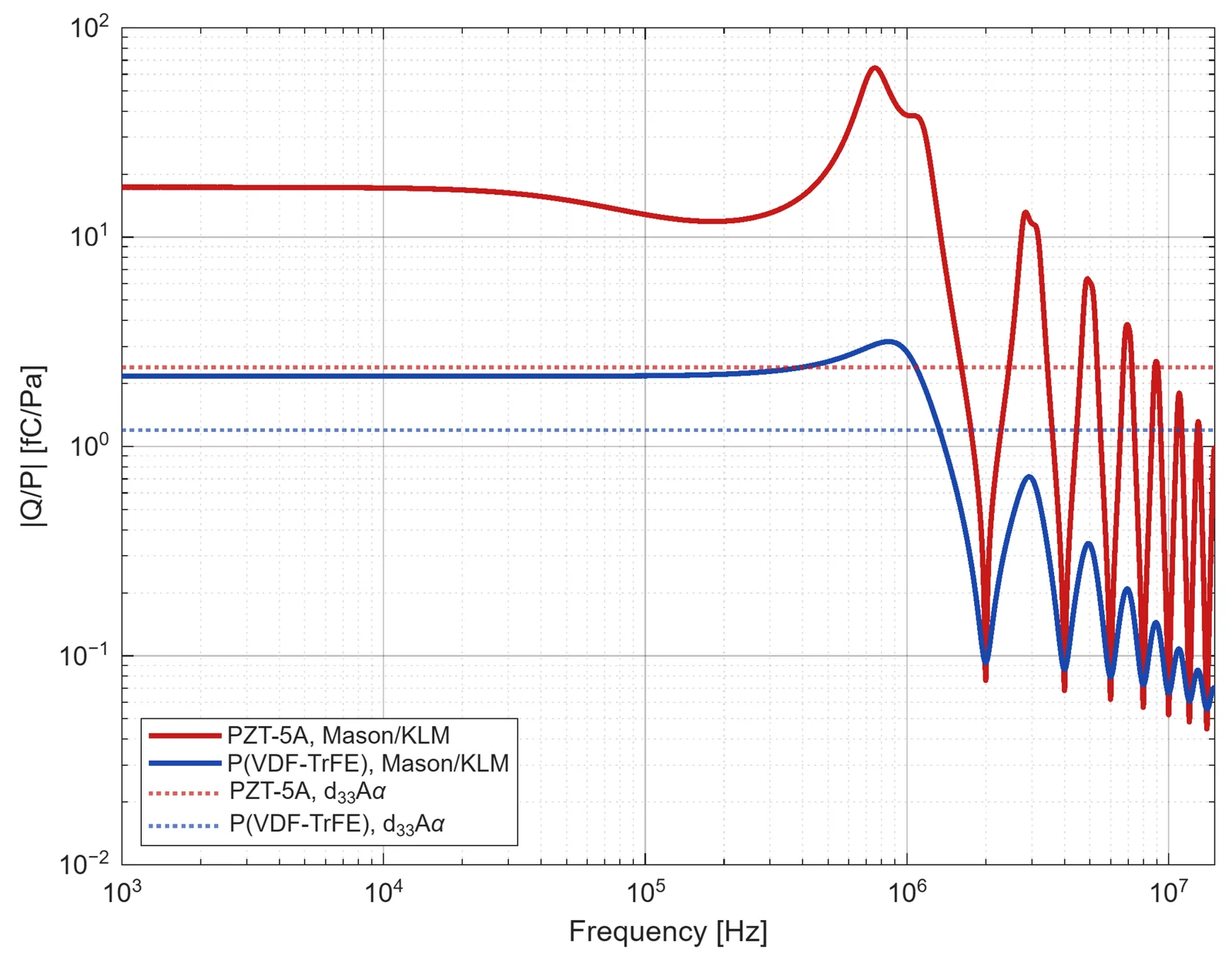
Short-circuit charge sensitivity from the distributed Mason/KLM model, compared with the quasi-static expression *d*_33_·A·*α* (dotted), which is widely used as a first estimate of hydrophone sensitivity. The two differ by 5 dB for the polymer and by 15 dB for the ceramic, as discussed in Section 2.3.

Figure 3 and Figure 4 show the output noise spectra of the two detectors broken down into their components, and Figure 5 the corresponding input-referred spectra. Figure 6 compares the charge sensitivity of the distributed model with the quasi-static expression of Section 2.3. Table 2 gives the integrated budget.

The two detectors are limited by different mechanisms. The copolymer budget is dominated by the sensor itself, and within it by the dielectric loss, with the radiation term second. The electronics add a noise figure of 0.61 dB, so the readout is practically noiseless. The PZT-5A budget is dominated by the electronics, which account for 115 of the 124 mPa rms, almost entirely through the amplifier voltage noise, giving a noise figure of 8.57 dB. The cause is the capacitance ratio of Section 2.2: the ceramic presents 384 pF to an amplifier whose voltage noise is amplified by the noise gain, while its charge sensitivity is only six times larger than that of the polymer.

The resulting advantage of the copolymer is 8.29 dB over 1 kHz–1 MHz and 8.58 dB over the 30–200 kHz processing band of Section 3.4. This figure is not a statement that the polymer is intrinsically quieter. As sensors alone the two are equivalent, 46.1 against 44.4 mPa rms, a difference of 0.3 dB, and with a noiseless amplifier they would perform alike. The advantage of the polymer is that its budget is insensitive to the readout, and the noise figures express this.

The narrow features at 56.1 and 467.5 kHz for PZT-5A and at 23.6 and 196.4 kHz for the copolymer are the in-plane branches of Section 2.5. They are sharp for the ceramic, whose intrinsic mechanical quality factor is 75, and shallow for the polymer, whose *Q_m_* is 10. The integrated contributions, 35.9 mPa rms for the ceramic against 4.9 mPa rms for the polymer, follow from the branch inductances, not from the quality factors, as noted in Section 2.5. The transverse coupling coefficient of the ceramic is seven times larger and its static capacitance fifty-six times larger, which together make its motional branches far more effective at delivering thermal charge to the amplifier.

### 3.2. Model Verification and Robustness

Because these results rest on a model and not on measurement, four independent checks were performed.

The first is a set of analytical limits. For a free plate with both faces unloaded the computed electrical impedance reproduces the closed form (1/j*ωC*_0_) [1 − *k_t_*^2^ tan(*θ*/2)/(*θ*/2)] to a relative error below 3 × 10^−15^. For an acoustically transparent element, a fictitious material whose impedance equals that of water, with water on both faces, the computed short-circuit charge sensitivity reproduces the quasi-static value to within the piezoelectric-stiffening correction, the agreement being 1.02. For the water-backed copolymer, which at low frequency is an acoustically thin plate loaded by water on both faces, the radiation noise obtained from the port conductance at 10 kHz is 12.86 µPa/√Hz, against the analytical √(2*k_B_*T*Z_w_*/A) = 12.87 µPa/√Hz. Noise and signal are thus referred to the same pressure.

The second is the quasi-static identity of Equation (3). The charge sensitivity obtained from the distributed model at 1 kHz agrees with (*e*_33_/*c*_33_*^E^*)·A·2*Z_b_*/(*Z_w_* + *Z_b_*) to within 0.2% for both materials and for four rear terminations between 1.5 and 10 MRayl, which verifies the formulation of Section 2.3 independently of the numerical implementation.

**Figure 7:**
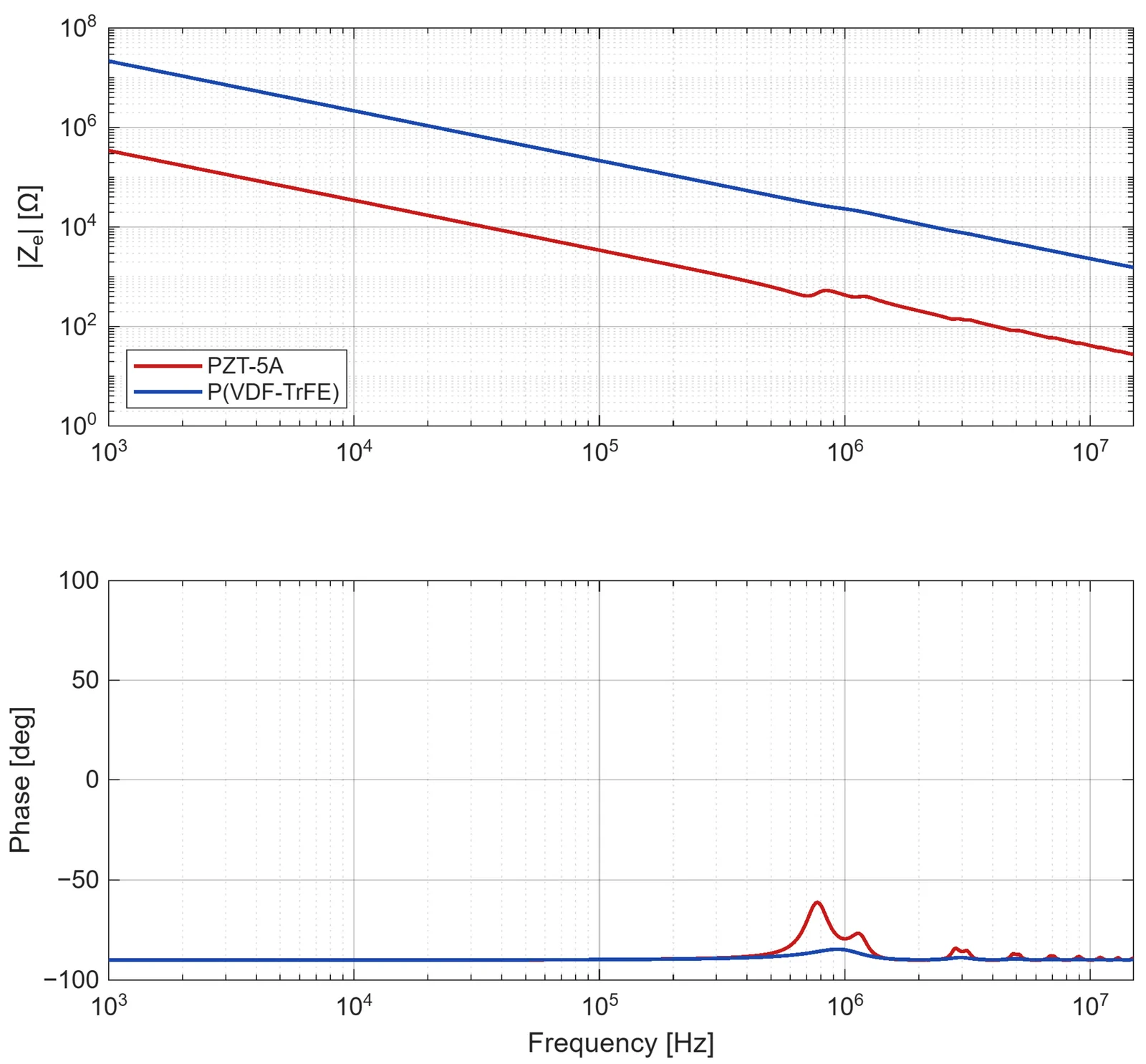
Magnitude and phase of the electrical impedance of the two elements, computed from the distributed Mason/KLM model.

The third is a cross-verification between the two levels of description, which also bounds the role that the lumped network is allowed to play. The electrical impedance obtained from the reduced network and from the distributed model, the latter shown in Figure 7, agree to a median of 1.6% over the integration band for the copolymer, whose *k_t_* is 0.30, and only to 18% for PZT-5A, whose *k_t_* of 0.49 exceeds the value of 0.4 below which the IEEE approximation for the motional capacitance is normally applied. The deviation is largest near the resonances, where the lumped branch places a single pole while the distributed model carries the loaded resonance together with its harmonics. This is why the signal path, the electrical impedance and the thermal noise of the thickness mode are taken from the distributed model throughout, and the lumped network is used only where a distributed description is unavailable, namely for the in-plane branches of Section 2.5, whose integrated contribution is in any case governed by their inductance, not by their damping.

**Figure 8:**
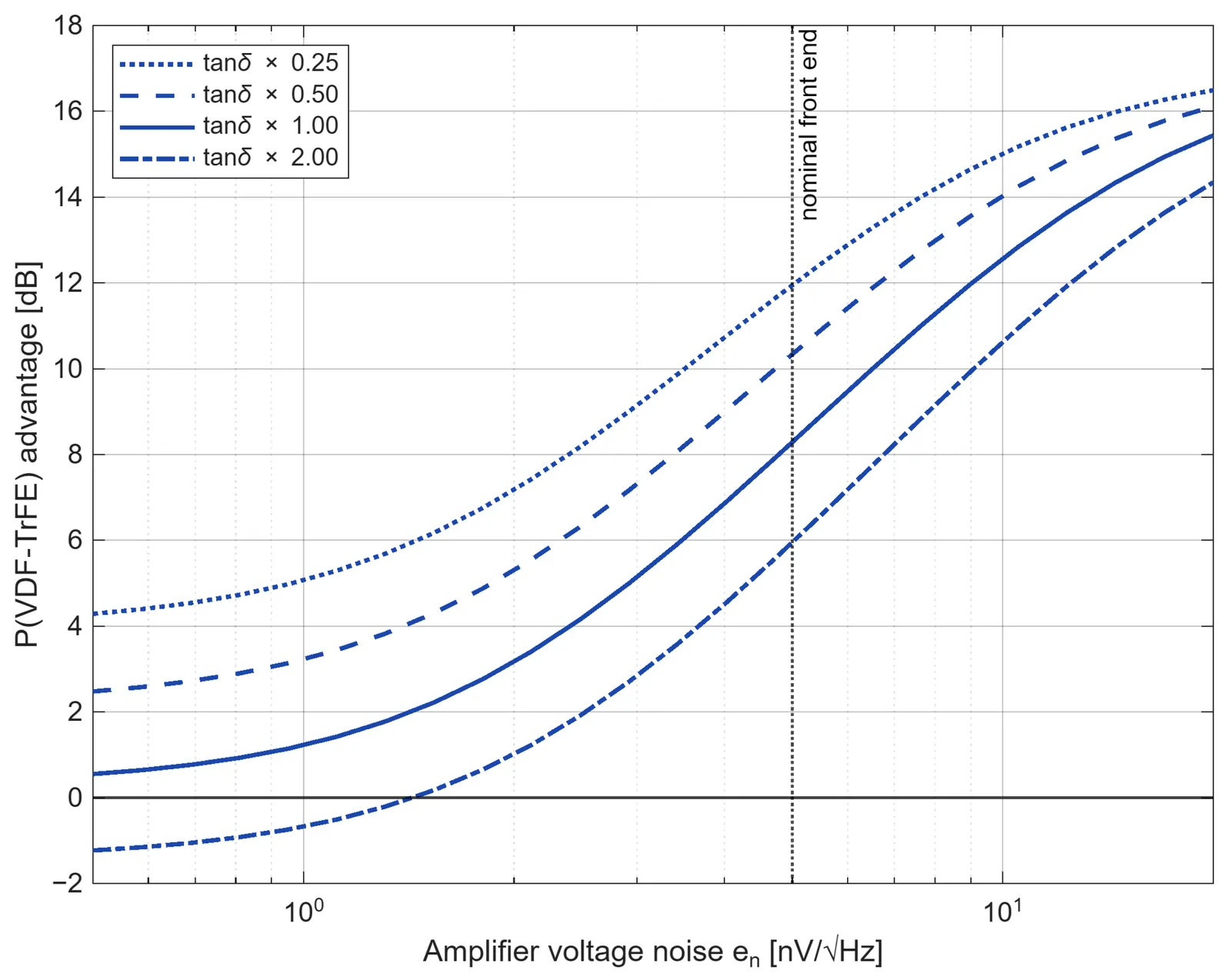
Advantage of the copolymer over PZT-5A in integrated noise-equivalent pressure, as a function of the amplifier voltage noise and of a uniform scaling of the polymer loss-tangent curve. The vertical line marks the nominal front end of Section 2.6. The advantage ranges from −1.2 to 16.5 dB, and it is negative only for a loss tangent twice the nominal curve combined with a voltage noise below 1.4 nV/√Hz.

The fourth is a parametric robustness analysis, reported in Figure 8. The two quantities that carry the largest uncertainty are the loss tangent of the polymer, whose frequency dependence is taken from measurements on a related material, and the voltage noise of the amplifier, which is a design choice. Scaling the whole loss-tangent curve by a factor of 0.25, 0.5 and 2 changes the advantage of the copolymer from 8.29 dB to 11.95, 10.34 and 5.95 dB respectively. A factor of eight in the least certain material parameter thus moves the conclusion by 6 dB, without reversing it at the nominal readout. Varying the amplifier voltage noise between 1 and 14 nV/√Hz changes the advantage from 1.23 to 14.20 dB. The low end is not hypothetical, since front ends below 1 nV/√Hz have been reported for this application [31]. With a readout of that class the two detectors approach parity, and if the polymer loss tangent is also twice the nominal curve the ordering reverses below 1.43 nV/√Hz, down to −1.23 dB at 0.5 nV/√Hz. The magnitude of the advantage, and in one corner of the map its sign, thus depend on the readout, which is the point developed in Section 4.3.

### 3.3. In-Band Amplitude and Phase

**Figure 9:**
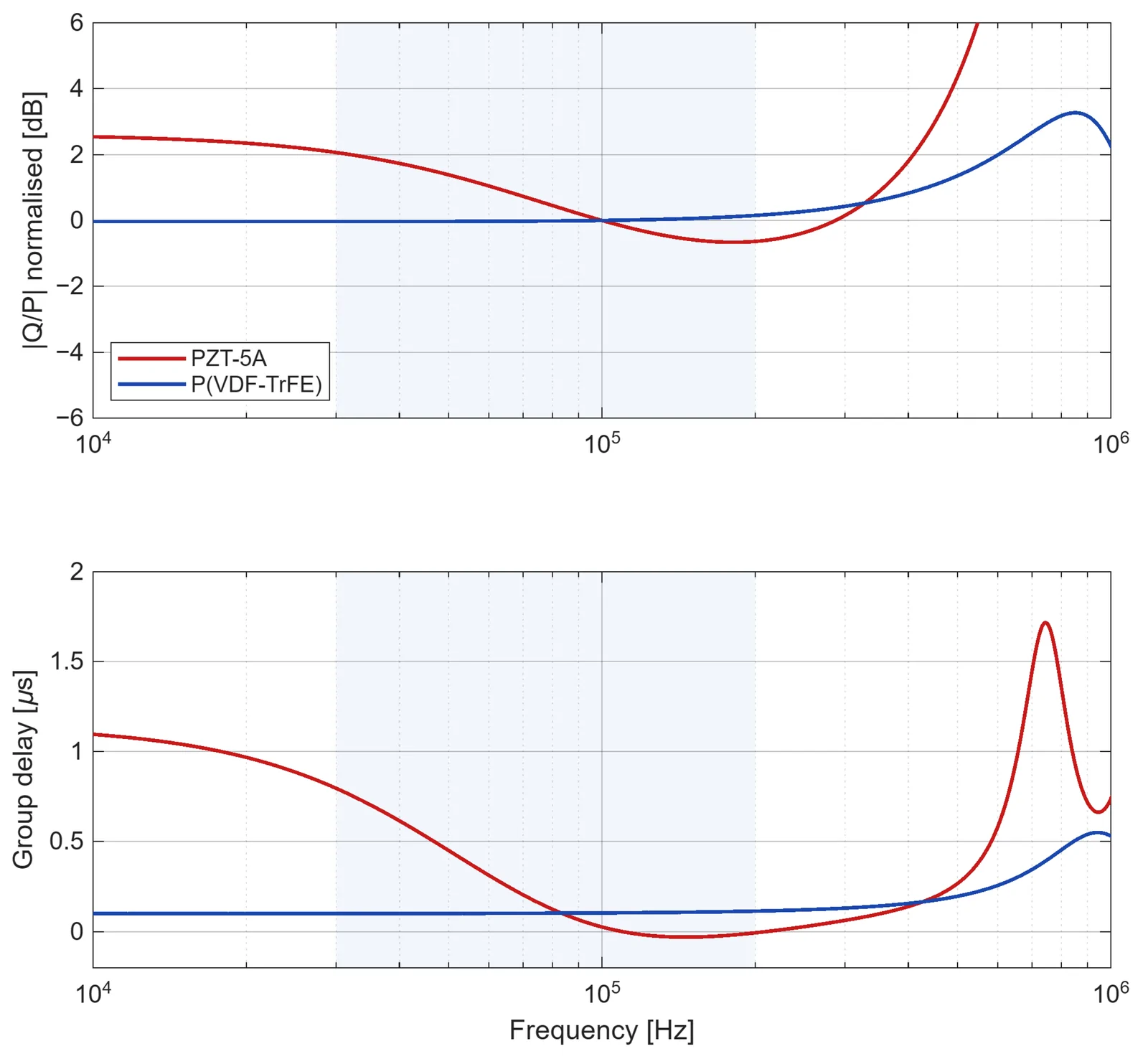
Normalised magnitude and group delay of the pressure-to-charge response across the ionoacoustic band. The backed ceramic varies by 2.73 dB and its group delay by 0.83 µs, equivalent to 1.24 mm of propagation in water; the water-backed copolymer varies by 0.19 dB and 0.014 µs. The shaded region is the 30–200 kHz processing band of Section 3.4.

The quantity that matters for range verification is not only the signal-to-noise ratio but the fidelity with which the arrival time of the acoustic transient is preserved, since it is the time of flight that encodes the Bragg-peak position. Figure 9 shows the magnitude and the group delay of the pressure-to-charge response across the band.

Over 30–200 kHz the response of the backed ceramic varies by 2.73 dB in magnitude, departs from a linear phase by up to 3.6°, and has a group delay that varies by 0.83 µs across the band, equivalent to 1.24 mm of propagation in water. The water-backed copolymer varies by 0.19 dB, departs from linear phase by 0.10°, and has a group-delay spread of 0.014 µs, equivalent to 0.021 mm. The ceramic is dispersive within the signal band, by an amount comparable with the clinical range-verification requirement, while the polymer is not.

This dispersion is deterministic and can in principle be removed by deconvolution, provided that the response is known. The practical distinction is one of robustness, not of principle: the resonance of the ceramic depends on temperature, on the bonding of the matching layer and on the backing, so that its correction requires a calibration to be established and maintained, whereas the polymer requires none. The imaging study of the next section is therefore performed without deconvolution, which is the processing chain of an experiment that applies only a band-pass filter.

### 3.4. Ionoacoustic Imaging and Range Accuracy

An acoustic simulation was performed with k-Wave [58] to compare the two materials in the reconstruction of a clinical proton beam at 200 MeV delivering 10 mGy at the Bragg peak. Because the simulation is two-dimensional, the corresponding dose per pulse in three dimensions is higher, 54 mGy, as derived below. The depth-dose profile was obtained from the analytical Bragg curve of Bortfeld [59]: the unstraggled distribution (*R*_0_ − z)^1/p−1^ with p = 1.77 and *R*_0_ = *α*E^p^ = 26.0 cm for 200 MeV in water was convolved with a Gaussian of standard deviation 0.36 cm representing range straggling and beam energy spread, which also removes the singularity at z = *R*_0_ [60,61]. The lateral profile was Gaussian with a standard deviation of 3 mm. The initial pressure follows as *p*_0_ = Γ*ρ* D with a Grüneisen parameter Γ = 0.11 for water at room temperature, giving 1.10 Pa at the Bragg peak.

The depth-dose profile is analytical and not Monte Carlo, and the cost of that choice can be bounded. Recomputing it with the complete Bortfeld curve, which adds the fluence reduction caused by nuclear interactions and the dose deposited by their secondaries (*β* = 0.012 cm^−1^, *γ* = 0.6, *ε* = 0.1), lowers the peak-to-entrance ratio from 6.6 to 4.6 and widens the peak from 2.25 to 2.39 cm. Yet it changes the source spectrum integrated over the 30–200 kHz processing band by 0.03 dB and displaces the 80% distal fall-off by 0.02 mm. Scaling the straggling width by 0.7 and by 1.3 changes the same quantity by +0.28 and −0.68 dB. The processing band corresponds to wavelengths between 7.5 and 50 mm in water, which is coarse compared with the sub-millimetre features in which an analytical depth-dose curve and a Monte Carlo transport calculation differ, and the source term is in any case common to the two materials, so that the comparison reported below is insensitive to it at first order. The Grüneisen parameter adopted here is that of water at room temperature; at body temperature it is about twice as large, so that the pressures, and with them the signal-to-noise ratios of this section, are conservative by approximately 6 dB.

**Figure 10:**
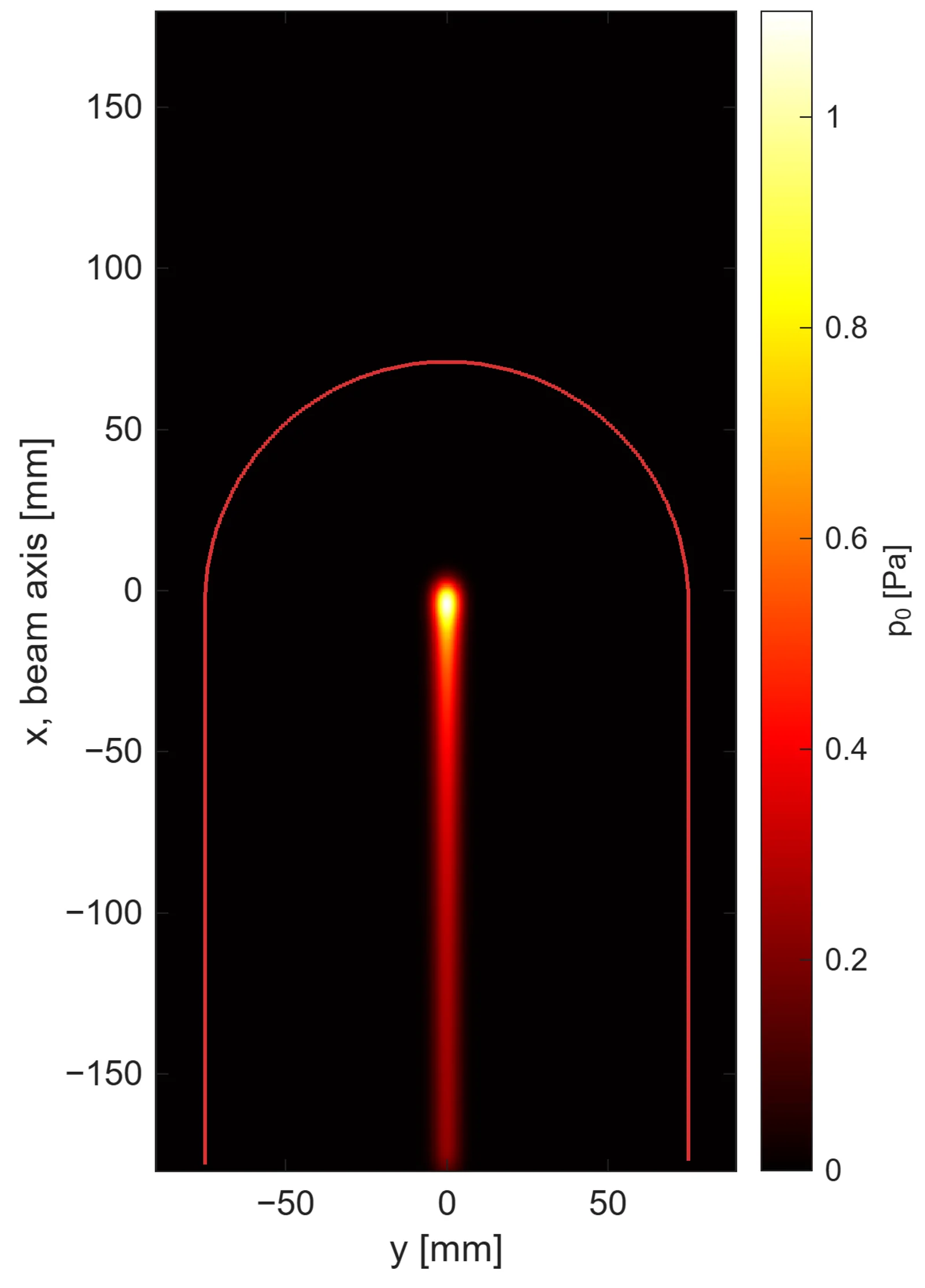
Initial pressure distribution of the 200 MeV proton beam in the simulation plane, at 10 mGy at the Bragg peak (54 mGy per pulse in three dimensions, Section 3.4), with the 512-element U-shaped aperture superimposed.

The computational domain was 360 × 180 mm on a Cartesian grid of 720 × 360 points with uniform spacing of 0.5 mm, in a homogeneous water medium. A perfectly matched layer of 20 grid points was applied on all boundaries and the time step was set by a CFL number of 0.3, giving a sampling rate of 10 MHz. The detector aperture is U-shaped at a constant stand-off of R = 75 mm from the beam axis: two straight sections parallel to the beam, joined on the distal side by a semicircular arc of radius R centred on the Bragg peak, with a total perimeter of 583 mm within the simulated domain (Figure 10). The element count follows from the spatial sampling requirement of the reconstruction, which demands a pitch not exceeding half the shortest wavelength in the signal band, that is N ≥ 2P*f_max_*/*c_w_* = 389 for *f_max_* = 500 kHz; the array comprises 512 elements with a pitch of 1.14 mm, which satisfies the criterion up to 659 kHz.

Noise was introduced in the input-referred pressure domain, each realisation being synthesised by shaping white Gaussian noise with the square root of the input-referred power spectral density of Section 2.6 over the 1 kHz–1 MHz band, independently between elements and between runs. The sum of signal and noise was then filtered by the complex pressure-to-charge response of the corresponding element, taken from the distributed model with its amplitude and phase, so that the recorded quantity is the charge that the element actually delivers; no deconvolution of the sensor response was applied. The traces were then band-pass filtered to 30–200 kHz, the band in which the ionoacoustic signal of a 200 MeV beam is concentrated, with a fourth-order Butterworth response applied forwards and backwards so that the group delay of the filter is zero and the acoustic time of flight is preserved [20,26]. The width of that band depends on the duration of the beam pulse as well as on the extent of the dose distribution, and it bounds the resolution of any radiation-induced acoustic image [62]. Images were reconstructed by time reversal as implemented in k-Wave [63].

The signal amplitude was taken as the mean reconstructed pressure over a 4 mm disc centred on the known position of the Bragg peak in the noiseless reconstruction of the same material, which separates the amplitude lost to the limited view of the partial aperture, common to both materials, from the effect of the sensor noise. The noise amplitude was taken as the standard deviation of the difference between the noisy and the noiseless reconstruction which, the reconstruction being linear, is exactly the reconstructed noise field; no background region has to be selected and the estimate is unbiased in every regime.

**Figure 11:**
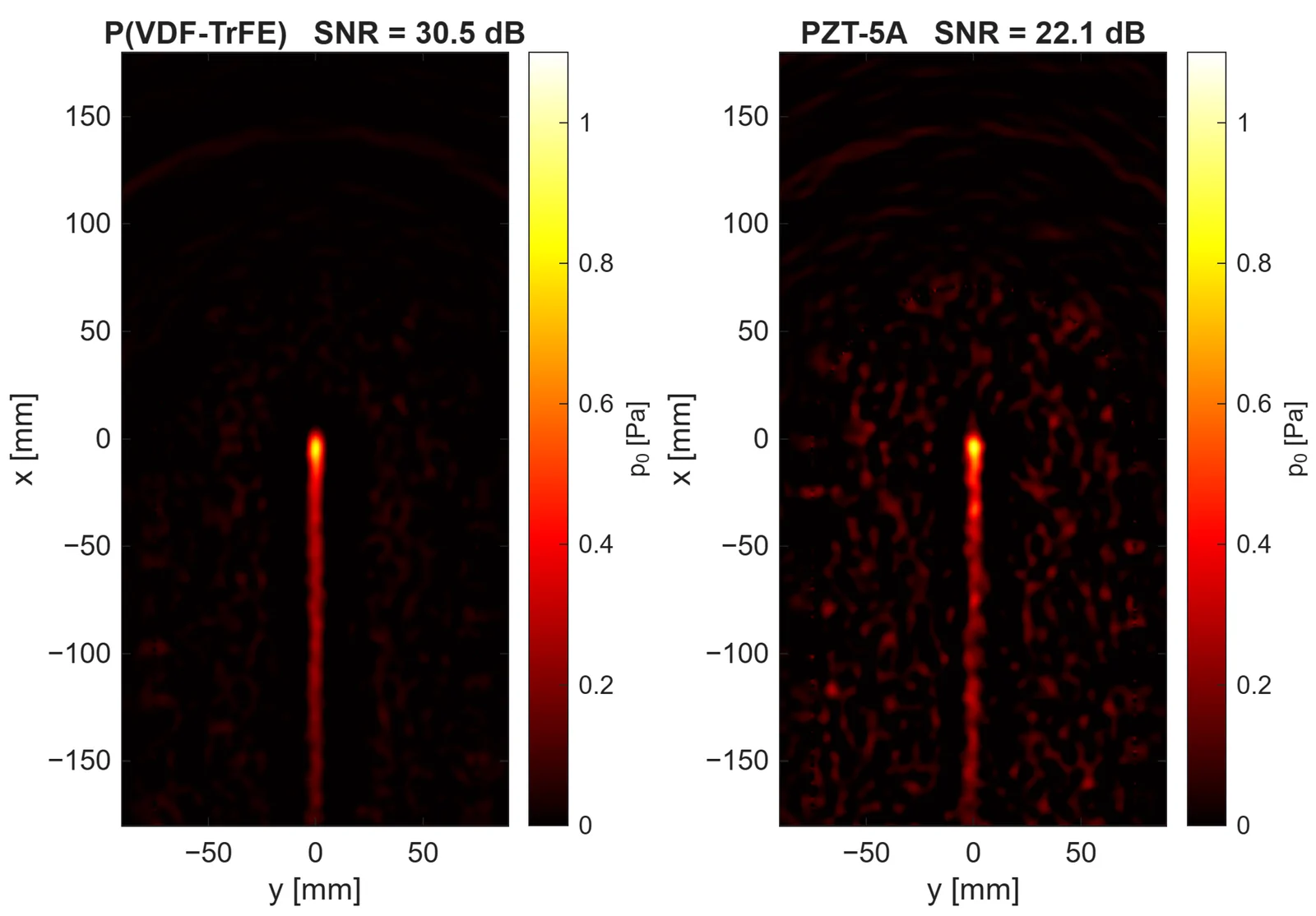
Single-pulse reconstructions obtained with the P(VDF-TrFE) and PZT-5A arrays, each element carrying the noise spectrum of Section 2.6 and the response of Section 3.3, with no deconvolution. Both panels share the same absolute colour scale.

Over five independent noise realisations the reconstructed signal-to-noise ratio (Figure 11) is 30.52 ± 0.22 dB for the copolymer array against 22.07 ± 0.25 dB for the PZT-5A array, a difference of 8.45 dB. This verifies that the imaging chain is consistent with the noise model, since the ratio of the two noise-equivalent pressures evaluated analytically over the same processing band is 8.58 dB: the imaging chain, which involves propagation over a partial aperture, the sensor response, band-limited filtering and time-reversal reconstruction, returns the same figure to within 0.13 dB. Both arrays localise the Bragg peak from a single pulse: the noise-driven localisation error, estimated from the displacement of the reconstructed axial profile with respect to the noiseless reconstruction of the same material, is 0.020 mm rms for the copolymer and 0.054 mm rms for PZT-5A, far below the clinical requirement. In experiments at pre-clinical energies, localisation errors of up to 1 mm have been traced to an imperfect knowledge of the sensor positions as well as to noise [64]. The figures above are thus a lower bound on the error of a real array.

**Figure 12:**
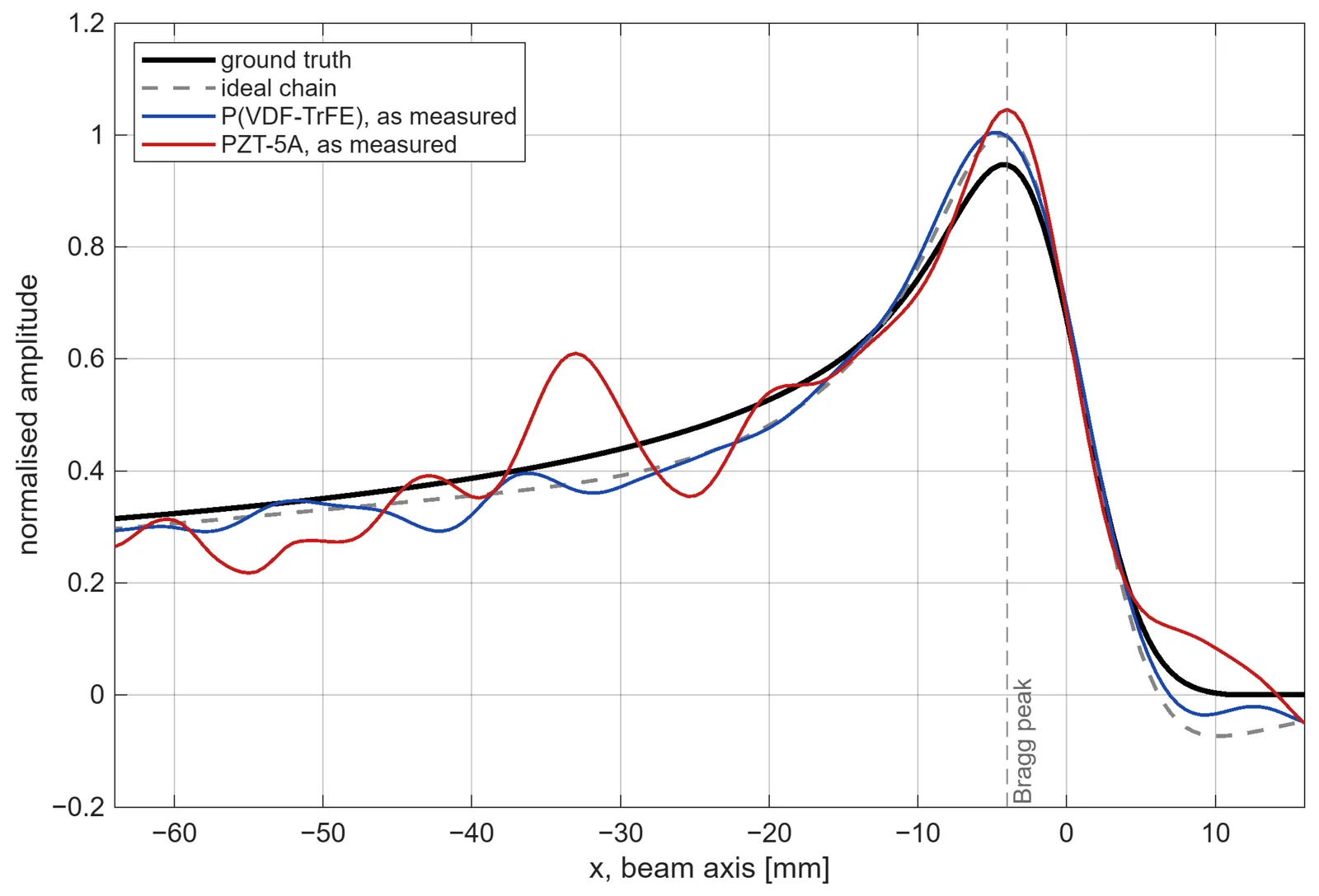
Reconstructed profile along the beam axis: ground truth, ideal chain with the sensor response deconvolved, and the two materials as measured. The displacement of the distal edge is the systematic range error discussed in the text.

The transducer response, however, contributes a systematic range error that the noise does not. Reconstructing the noiseless traces after filtering them by the element response shifts the apparent Bragg peak by 0.058 mm for the copolymer and by 0.334 mm for PZT-5A, with respect to the ideal chain in which the response is perfectly deconvolved (Figure 12); the residual offset of the ideal chain itself, −0.387 mm, is the limited-view bias of the U-shaped aperture and is common to both. The ceramic carries a range bias nearly six times larger, and one which depends on the stability of its own resonance, in exchange for a signal-to-noise ratio 8.45 dB lower. Raising the signal-to-noise ratio beyond the single-pulse value requires processing that rejects the random noise in favour of the deterministic waveform, as in the correlation-based evaluation of ionoacoustic traces [26], the deep-learning denoising of radiation-induced and protoacoustic signals [65,66] and the real-time spike detectors developed for neural interfaces [67,68]. Range has been verified from single protoacoustic pulses at a clinical synchrocyclotron [69], and extracting the arrival time is a digital task for the converters and filter chains developed for detector and sensor readout [55,56].

The simulation is two-dimensional, and a two-dimensional source is uniform along the axis normal to the simulation plane. The modelled beam is not: its transverse profile is Gaussian with the same standard deviation, *σ* = 3 mm, in both directions, so the field it radiates is weaker. Since the medium is homogeneous, the correction can be obtained in closed form. Integrating the free-space Green’s function across the beam in the paraxial approximation, for a receiver at distance r from a source point in the simulation plane, the ratio between the three-dimensional and the two-dimensional field is

(7) $$H\left(f,r\right)=\frac{\sigma \sqrt{\frac{k}{r}}}{\sqrt{1-\frac{jk\sigma^{2}}{r}}}e^{-j\pi /4}$$

where k = 2πf/c. The expression has the expected limits: |H| tends to *σ*√(k/r) for a beam narrow compared with the Fresnel zone √(r/k), and to 1 for a beam wide compared with it, where the two geometries coincide. At r = 75 mm it amounts to −18.2, −13.0 and −10.0 dB at 30, 100 and 200 kHz. Equation (7) was applied to each element, with r its distance from the Bragg peak and the trace gated around the arrival of the Bragg-peak signal, and weighted by the signal spectrum and by the input-referred noise of each detector over the processing band. The resulting effective attenuation is 14.7 dB for the copolymer and 15.2 dB for PZT-5A; without the gate both figures change by less than 0.5 dB. Since the pressures are linear in the dose, the signal-to-noise ratios obtained in the simulation at 10 mGy are reached in three dimensions at 54 mGy per pulse. At that dose the copolymer array reaches the 30.5 dB of the simulation and PZT-5A reaches 21.5 dB: because the noise of the copolymer falls with frequency, its signal-to-noise ratio draws more on the upper part of the band, where the correction is smaller. The difference thus becomes 9.0 dB. The two-dimensional values, which are the conservative ones for the comparison, are those quoted in the rest of this paper. A full three-dimensional acquisition is feasible with matrix arrays: one of 256 elements has tracked the Bragg peak in real time on a human torso phantom at a clinical proton machine [70].

The difference in signal-to-noise ratio can be translated into a dose equivalence, but only under stated conditions. If the dose per pulse is held fixed and the noise is statistically independent between successive pulses, coherent averaging of N pulses improves the ratio as √N, so that a deficit of 8.45 dB corresponds to N ≈ 7 pulses, that is a total delivered dose of about 380 mGy against the 54 mGy required by the copolymer array for equivalent image quality. Three caveats bound this figure: it assumes ideal coherent averaging, whereas pulse-to-pulse variations in beam intensity, energy and position introduce a residual incoherent component; it assumes noise uncorrelated between pulses, which excludes drift synchronous with the accelerator cycle; and it holds the dose per pulse fixed, so it does not describe a regime in which pulse intensity can be raised instead.

### 3.5. Distance from the Thermal Floor

The acoustic radiation floor of the front face is the only term in the budget that cannot be reduced by any choice of material or readout. For the 75 mm^2^ aperture considered here it amounts to *k_B_*T*Z_w_*/A = 9.10 µPa/√Hz, that is 9.1 mPa rms over the 1 kHz–1 MHz band, against integrated totals of 47.6 mPa rms for the copolymer and 123.7 mPa rms for PZT-5A. The copolymer detector operates 14.4 dB above the thermal limit of its own aperture, and the ceramic 22.7 dB above it.

This has a direct design consequence. For the copolymer, the margin available to any improvement of the readout is at most 0.61 dB. The remaining headroom lies in the dielectric loss of the polymer: removing it altogether would lower the total from 47.6 to 22.6 mPa rms, a gain of 6.5 dB, beyond which the radiation of the two faces and the electronics set the limit. Material development is thus the productive direction, and the self-polarised and composite formulations now being reported [71] should be judged by their loss tangent, not only by their piezoelectric constant. For PZT-5A the headroom lies in the electronics, and capturing it requires a front end whose voltage noise is of the order of 1 nV/√Hz while driving 384 pF.

## 4. Discussion

### 4.1. Interpretation of the Results

For a piezoelectric receiver operating in water near its noise floor, the relevant figure of merit is neither the piezoelectric charge constant nor the acoustic impedance alone, but the charge sensitivity of Section 2.3 divided by the capacitance that the readout must drive. Under this criterion the two materials examined here reach comparable sensor-limited performance by opposite routes, and the system-level ordering is decided by the readout.

The ceramic has the higher charge sensitivity, 12.8 fC/Pa against 2.18 fC/Pa at 100 kHz, and the lower dielectric loss, and as a sensor alone it is equivalent to the polymer, within 0.3 dB. What it cannot escape is its permittivity: 384 pF of clamped capacitance multiply the amplifier voltage noise through the noise gain, and 115 of its 124 mPa rms are contributed by that single term. The polymer, with 6.9 pF, is immune to the same term, and its budget is set by its own dielectric loss. The practical consequence is that the ceramic is only as good as the amplifier in front of it, while the polymer is as good as the material allows regardless of the amplifier.

### 4.2. Implications for Polymer–Ceramic Comparisons

The distinction drawn in Section 2.3 has implications beyond the present comparison. The expression *S_q_* = *d*_33_·A·*α* is a convenient first estimate of hydrophone sensitivity. For a polymer immersed in water it falls within about 5 dB of the distributed result, since the material constant is almost unchanged and the transmission term is moderate. For a stiff ceramic the transmission term prevails, and the expression underestimates the charge sensitivity by 14–17 dB in the geometry considered here. A polymer–ceramic comparison based on it therefore favours the polymer by roughly the difference, about 10 dB. Evaluated in that way, the configuration studied here returns an advantage of 18.2 dB over the processing band, against 8.6 dB with the distributed model. The advantage that remains arises from a different mechanism, namely the capacitance and its interaction with the front end. That the ordering survives the change in description is not a foregone result, and it survives for a reason unrelated to the one the quasi-static estimate would suggest.

### 4.3. Design Implications: Material and Front End Are Not Separable

Figure 8 presents the comparison as a design map instead of a single number. Two parameters determine the outcome: the loss tangent of the polymer, which sets the floor of the polymer detector, and the voltage noise of the amplifier, which sets the floor of the ceramic detector. Within the ranges examined the advantage of the copolymer varies from −1.2 dB to 16.5 dB, and it is negative only where a loss tangent twice the nominal curve meets a voltage noise below 1.4 nV/√Hz.

Four practical statements follow. First, at the nominal loss tangent the ceramic does not overtake the polymer by improving the amplifier alone: with a front end of 1 nV/√Hz it remains 1.2 dB behind, and 0.6 dB at 0.5 nV/√Hz, because at that point its own sensor noise, and within it the in-plane branches, has become the limit. The two are then practically equivalent, and the ceramic moves ahead only if the polymer loss tangent is also higher than assumed. Second, the two materials call for different amplifier classes, the ceramic for low voltage noise driving 384 pF and the polymer for femtoampere bias currents and minimal input capacitance, so that a comparison performed with a single amplifier favours whichever material that amplifier suits. Third, the polymer requires an integrated front end, not for its noise but for its feedback network: a gigaohm resistance across 0.69 pF, with an amplifier input capacitance comparable to the element capacitance itself, is not a printed-circuit design. Fourth, the in-plane resonances depend on the lateral dimensions alone. A receiver required to be flat across the ionoacoustic band should be subdivided in the narrow dimension and not thinned, since reducing the element width below *c_bar_*/(2*f_max_*) displaces the width mode above the band while the aperture, and therefore the acoustic floor, is preserved by connecting several strips in parallel. This leaves the length mode in place, at 56.1 kHz for the ceramic, and moving it would require shortening the element along the beam axis. Thinning, by contrast, raises the thickness resonance but leaves the in-plane modes untouched and degrades the input-referred noise, because *C*_0_ scales as 1/t while the charge sensitivity does not.

### 4.4. Limitations

The scope of these results is bounded in six respects. None of them reverses the comparison, and where the model itself allows the effect to be bounded, the bound is given. First, there is no direct experimental validation on the two specific elements analysed. The individual components of the model are standard: the Mason and KLM descriptions are the established models of the thickness mode [44,48,49], and the thermal noise of each loss follows from the fluctuation–dissipation theorem [2,3,4]. What is new is their combination, and Section 3.2 verifies it against analytical limits, against the quasi-static identity of Equation (3) and across the two levels of description. A dedicated campaign, comprising electrical impedance spectroscopy on both materials, from which *k_t_* and the loss tangent can be extracted directly, and a calibrated reciprocity measurement of the receiving response, is in progress and will be reported separately.

Second, two parameters of the copolymer are taken from sources that do not correspond exactly to the modelled film. The thickness coupling coefficient is the minimum value quoted by the supplier, consistent with the classical characterisation of this composition [39,40], and the frequency dependence of the loss tangent is measured on copolymer blends [57]. Both effects are bounded. Scaling the loss-tangent curve between 0.25 and 2 times its nominal value keeps the advantage of the copolymer between 6.0 and 12.0 dB (Section 3.2). The coupling coefficient would have to fall to about 0.12, 40% of the minimum quoted by the supplier, before the conclusion changed.

Third, the treatment remains essentially one-dimensional. The thickness mode is treated exactly and the two in-plane extensional modes are included as additional branches, but the coupling between modes is not captured. A three-dimensional finite-element analysis would be needed to determine how strongly the in-plane modes receive the incident field. Shear and flexural modes, weakly excited at normal incidence, are not represented. The present assumption, that the in-plane branches dissipate without receiving, penalises the ceramic in the noise budget, but only slightly. Removing the in-plane branches from the budget altogether lowers the ceramic total from 123.7 to 118.4 mPa rms and the polymer total from 47.6 to 47.4 mPa rms, so that the advantage of the copolymer moves from 8.29 to 7.95 dB. The same assumption protects the ceramic in Section 3.3, because a mode at 56 kHz with a quality factor of 75 that did couple to the incident field would distort the waveform far more than the thickness resonance does.

Fourth, the model treats the sensor and the amplifier but not the interconnection. A printed-circuit parasitic at the summing node acts on the polymer channel like an increase in the amplifier input capacitance, and Section 2.6 quantifies a pessimistic case: 10 pF, about fourteen times *C_f_*, raises the NEP of the copolymer by 1.5 dB and that of PZT-5A by 0.2 dB, which still leaves an advantage of 6.9 dB. This is why Section 2.6 treats the integrated front end as a requirement for the polymer channel.

Fifth, the conclusions are established for the two specific designs analysed. The mechanisms responsible, namely the capacitance ratio and the loss tangent, are not specific to this geometry, but the quantitative figures should not be transferred to other dimensions without re-evaluation. The deposited code allows that re-evaluation to be carried out directly for other geometries and materials.

Sixth, the noise realisations of Section 3.4 are drawn independently between elements. That is correct for the amplifier, the feedback resistor, the dielectric loss and the in-plane branches, which are local to each channel, but not for the acoustic radiation term, which is the thermal field of the medium itself and whose spatial coherence is sinc(kd): at the 1.14 mm pitch of the array it is 0.96 at 100 kHz. A coherent component of this kind is not suppressed by the reconstruction in proportion to the number of elements, since its rejection is bounded by the directivity of the aperture and not by √N. The agreement between the reconstructed and the analytical signal-to-noise ratios in Section 3.4 is thus a verification of the processing chain, not an independent confirmation of the noise budget. The effect is bounded: in the limiting case in which the reconstruction does not reduce the radiation term at all, the imaging advantage of the copolymer falls from 8.45 dB towards 2.7 dB, the ratio of the two radiation terms over the processing band. Across these six points the advantage of the copolymer never falls below 6.0 dB in the integrated NEP, at twice the nominal loss tangent; in the imaging study the lower bound is 2.7 dB, reached only if the reconstruction did not reduce the radiation term at all. Its dependence on the readout is a design choice and not a limitation of the model, and is the subject of Section 4.3.

### 4.5. Future Work

Future work will address the experimental validation described above, the three-dimensional modal analysis required to resolve the in-plane mode coupling, and the extension of the model to array configurations in which inter-element acoustic and electrical crosstalk becomes relevant. On the readout side, the arrival-time extraction of Section 3.4 lends itself to a real-time FPGA implementation of the kind already used for neural spike detection [67].

## 5. Conclusions

A frequency-domain noise model for immersed bulk piezoelectric hydrophones read out by a charge amplifier has been presented. The transducer is described by a distributed Mason/KLM model, which provides the electromechanical response, the matching-layer stack and, through the Nyquist theorem at its electrical port, the thermal noise of the thickness mode, and by a multi-branch Butterworth–Van Dyke network, which adds the in-plane extensional modes and keeps each loss mechanism to a single thermal noise term.

The charge sensitivity of an immersed plate has been shown to depart from the quasi-static form *d*_33_·A·2*Z_w_*/(*Z_w_* + *Z_c_*) whenever the element is laterally constrained and acoustically thin: the thickness-mode constant is then *e*_33_/*c*_33_*^E^* and the transmitted stress is set by the rear termination. The two departures act in opposite directions. For a stiff ceramic the second prevails and the quasi-static form underestimates the sensitivity by 14–17 dB, while for a polymer close to water in impedance the difference is about 5 dB.

With the distributed formulation, a P(VDF-TrFE) 75/25 receiver of 75 mm^2^ aperture achieves an integrated noise-equivalent pressure of 47.6 mPa rms over 1 kHz–1 MHz against 123.7 mPa rms for a PZT-5A receiver of identical geometry. As sensors the two are within 0.3 dB of each other; the 8.3 dB difference at system level is due to the capacitance of the ceramic, which amplifies the amplifier voltage noise and leaves it with a noise figure of 8.6 dB against 0.6 dB for the polymer. With a front end of about 1 nV/√Hz the difference falls to about 1 dB, so that material and readout have to be chosen together. In a k-Wave study of a 200 MeV proton beam at 54 mGy per pulse, recorded without deconvolution of the sensor response, the copolymer array reaches a reconstructed signal-to-noise ratio of 30.5 dB against 22.1 dB, and contributes a systematic range error of 0.06 mm against 0.33 mm. The copolymer detector operates 14.4 dB above the thermal floor of its own aperture, and its remaining headroom lies in the loss tangent of the polymer and not in the readout. These figures are specific to the two designs analysed; the mechanisms behind them are not.

## Figures and Tables

**Table 1:** Material constants used in the noise model. PZT-5A values are from Morgan/TRS datasheets and IEEE Std 176-1987 [37,38]; P(VDF-TrFE) 75/25 values are from the supplier data sheet [39], with *k_t_* taken as the quoted minimum coupling factor, consistent with the classical characterisation of this composition [40]. The clamped permittivity is quoted directly, not derived from the free permittivity, since the relation between them involves *k*_33_ and not *k_t_*. The acoustic impedance *Z_a_* and the thickness t are derived quantities. In-plane constants used for the lateral branches of Section 2.5 are *s*_11_*^E^* = 16.4 pm^2^/N and *k*_31_ = 0.344 for PZT-5A, and *Y*_1_*^E^* = 2.5 GPa and *k*_31_ = 0.05 for the copolymer, whose transverse coefficients satisfy *d*_31_ = *d*_32_ < 5 pC/N [39].

Parameter	Symbol	Unit	PZT-5A	P(VDF-TrFE) 75/25
Density	*ρ*	kg m^−3^	7750	1800
Open-circuit speed of sound	*c_D_*	m s^−1^	4350	2300
Piezoelectric charge constant	*d*_33_	pC N^−1^	374	30
Thickness-mode constant	*e*_33_/*c*_33_*^E^*	pC N^−1^	144	29
Clamped relative permittivity	*ε^S^*_33_/*ε*_0_	—	830	8.25
Mechanical quality factor	*Q_m_*	—	75	10
Dielectric loss tangent	tan *δ*e	—	0.020	0.01–0.22 (Section 2.6.3)
Acoustic impedance	*Z_a_*	MRayl	33.7	4.14
Thickness at *f*_0_ = 1 MHz	t	mm	2.17	1.15
Electromechanical coupling coefficient	*k_t_*	—	0.49	0.30
Rear termination	*Z_b_*	MRayl	6.0	1.5

**Table 2:** Integrated noise-equivalent pressure over 1 kHz–1 MHz, in mPa rms, with the charge sensitivity of Section 2.3 and the noise sources and amplifier of Section 2.6. The PVDF homopolymer is included for reference, with the same loss-tangent curve.

	Radiation (mPa)	Internal (mPa)	Dielectric (mPa)	In-Plane (mPa)	Electronics (mPa)	Total (mPa)	Sensor Only (mPa)	NF (dB)
PZT-5A	16.8	1.9	23.5	35.9	114.8	123.7	46.1	8.57
P(VDF-TrFE)	13.7	1.2	41.9	4.9	17.3	47.6	44.4	0.61
PVDF homopolymer	13.6	1.2	94.2	19.1	42.4	106.0	97.1	0.76

## Data Availability

The MATLAB code of the noise model, the distributed-parameter verification, the robustness analysis and the k-Wave imaging study, together with the simulated data from which every figure is generated, are openly available in Zenodo at https://doi.org/10.5281/zenodo.23049493.
